# Emergence phenology, uncertainty, and the evolution of migratory behavior in *Anax junius* (Odonata: Aeshnidae)

**DOI:** 10.1371/journal.pone.0183508

**Published:** 2017-09-08

**Authors:** Michael L. May, John A. Gregoire, Suzanne M. Gregoire, Maria Aliberti Lubertazzi, John H. Matthews

**Affiliations:** 1 Department of Entomology, Rutgers University, New Brunswick, NJ, United States of America; 2 Kestrel Haven Avian Migration Observatory, Burdett, NY, United States of America; 3 Concentration in History, Philosophy and the Social Sciences, Rhode Island School of Design, Providence, RI, United States of America; 4 Alliance for Global Water Adaptation, Corvallis, OR, United States of America; USDA Agricultural Research Service, UNITED STATES

## Abstract

Mass migrations by Odonata, although less studied than those of Monarch butterflies and plague locusts, have provoked comment and study for many years. Relatively recently, increasing interest in dragonflies, supported by new technologies, has resulted in more detailed knowledge of the species involved, behavioral mechanisms, and geographic extent. In this paper we examine, in four independent but complementary studies, how larval habitat and emergence phenology interact with climate to shape the evolution of migratory strategy in *Anax junius*, a common species throughout much of the eastern United States and southern Canada. In brief, we argue that fish predation on larvae, coupled with the need for ample emergent vegetation for oviposition and adult eclosion, dictates that larval development and survival is optimal in ponds that are neither permanent nor extremely ephemeral. Coupled with annual variation in regional weather and winters in much of their range too cold for adult survival, conditions facing newly emerged *A*. *junius* may unpredictably favor either local reproduction or long-distance movement to more favorable areas. Both temperature and hydroperiod tend to favor local reproduction early in the adult activity period and migration later, so late emerging adults are more likely to migrate. No single pond is always predictably suitable or unsuitable, however, so ovipositing females also may spread the risk to their offspring by ovipositing at multiple sites that, for migrants, may be distributed over very long distances.

## Introduction

Although insect migration has not received as much popular or scientific attention as migration of birds or large mammals, it is a biologically widespread and important phenomenon, with consequences for community structure and interactions, biomass and nutrient transfer, and vectoring of disease and ecological community interactions [1, 2]. Moreover, comparison of genetic structures of migratory and non-migratory populations have shed sometimes surprising light on their biogeography and demography [3]. Here we present new data on an important but understudied North American dragonfly and use these data to infer how selection has shaped its life history to include sometimes spectacular but facultative migration.

The Common Green Darner, *Anax junius* (Drury, 1770), is an abundant and conspicuous dragonfly throughout much of temperate North America, with adults occurring as far north as 50°N and larvae overwintering to at least 45°N [4]. Adult females, usually accompanied by a male in tandem, oviposit in a variety of aquatic habitats, ranging from longer-lasting rain pools to large lakes and occasionally slow-flowing canals or streams [5] (MLM, JHM, pers. obs). Most often, however, successful reproduction takes place in fairly shallow ponds with ample emergent or floating, non-woody vegetation available for oviposition [6, 7, 8]. Adults mate and oviposit at lakes or ponds containing fish (MLM, pers. obs., P. Morin, pers. comm., 2016), but larval survival and emergence is much higher if insectivorous fish, especially *Lepomis* spp. [9] are absent [6, 10, 11, 12], and populations are known to have been extirpated completely from breeding sites after introduction of fish [12] (D. M. Johnson, pers. comm., email, 2016; P. Morin, pers. comm., email, 2016).

Larvae may enter diapause (*sensu* Corbet) [13] and overwinter in these habitats except, perhaps, at the northern fringes of their range [14] and in the far south, where development is probably continuous. Alternatively, many undergo direct development and the adults may migrate in the fall many hundreds of kilometers south from their natal ponds before reproducing [15, 16]. Extensive annual migrations by *A*. *junius* in eastern North America have been documented repeatedly [16, 17, 18, 19]. A similar pattern has been noted in the West, especially the Pacific coastal states [8, 20] and across the southern plains [21, 22]. Russell, et al.[18] compiled a list of these accounts, summarizing the results as follows: “Records of large dragonfly migrations show several distinct patterns: (1) all reports fell between late July and mid-October, with a peak in September; (2) most of the large flights occurred along topographic leading lines such as coastlines and lakeshores; (3) massive swarm migrations generally followed the passage of synoptic-scale cold fronts; and (4) the common green darner (*Anax junius*) was the principal species involved in the majority of these flights.” Spring migration is less well documented, but ample evidence indicates that it also is an annual event [18, 19].

Of roughly 30 species of *Anax* (including *Hemianax*) worldwide, Corbet [13] listed 9 as known migrants. Most of these are primarily tropical and probably migrate with the Intertropical Convergence Zone (ITCZ), assuring that they track the associated latitudinal band of rains and thus find suitable habitat for oviposition and larval survival (a pattern which has been observed in greater detail in the libellulid *Pantala flavescens*, [23]). Whether or not most *Anax* exhibit larval diapause is unknown but is unlikely in strictly tropical/subtropical species. In these habitats temperatures typically permit adult activity and larval feeding year round, and in seasonal environments the dry season is usually passed as an adult or egg by species that reproduce in lentic environments, although some breeding in permanent waters might be exceptions [13, 24].

*Anax imperator*, one of the few other species that regularly breeds as far north as *A*. *junius*, ranges from northern Europe to southern Africa and has a larval diapause in its European range, but those populations do not migrate; populations in sub-Saharan Africa may migrate [13], but their diapause status is unknown. *Anax p*. *parthenope* (Selys, 1839) is a migrant and breeder in northern Eurasia, that is known to be semivoltine in northern portions of its range and bivoltine in the south, indicating that larval diapause has evolved in northern populations of this species. It is likely that the propensity of *Anax junius* to migrate is, in part, a legacy from ancestors that were tropical migrants, but only in *A*. *junius* and *A*. *parthenope* is there evidence of both facultative larval diapause and adult migration [16].

Quantitative studies of emergence and migration in *Anax junius* began with the work of Robert Trottier [14, 25] in Canada. He found that in southern Ontario (~43.5°N), two clear-cut cohorts of larvae existed, corresponding to adults with very different behaviors. Combined with observations of adults ovipositing in April, long before local emergence [6, 26], these data led Trottier to infer [14] that the first group of larvae represented “residents” that overwintered as mid- to late-instar larvae, grew to full size as waters warmed in spring to early summer, and emerged in midsummer. The adults matured, oviposited in or near their natal pond, and died by mid-August; their offspring hatched and grew to middle instar larvae before entering diapause for the winter. The second cohort were the offspring of adults that migrated into the area in early spring and had been seen ovipositing as early as April, sometimes with snow still on the ground [26]. These larvae grew rapidly in warm water in summer and matured by late summer, when they emerged as adults. Most of the latter left the vicinity while still sexually immature and migrated southward; presumably some of their offspring returned northward the next spring, and the “migrant” cycle began again.

Wissinger [27] reported a similar pattern of emergence from a population of *A*. *junius* in Indiana, although a few adults emerged very early, in April. He interpreted these as individuals from the previous year’s migrant cohort that had not completed development in time to emerge the previous fall and had diapaused over the winter in a late instar. This observation suggests that larval diapause is facultative, and it raises the possibility that a few early adults in northern localities may emerge locally (and see [28]). Kime [20] also reported migrant and resident cohorts of *Anax junius* larvae in Washington State, based on larval size distribution.

Trottier’s model suggested that migrants and residents are behaviorally and physiologically distinct. The two cohorts seemed reproductively isolated because the mating and oviposition periods of their respective adults did not overlap. The likelihood of genetic divergence and even incipient speciation of migrants and residents had to be considered.

More recent data, however, calls this scenario into question. Freeland, et al. [29] and Matthews [15, 30] found no genetic distinction between supposed resident and migrant populations. Matthews [31] (see also [19]) also revisited Trottier’s original study area in 2003–04 and found that, although two peaks of activity were apparent, adult flight periods overlapped and would allow for genetic exchange between early and late emergers. Thus, although migration appears to be a regular part of the life cycle of a sizable fraction of individuals of *A*. *junius*, it apparently is not based on genetic differences among populations.

In any event, migration clearly requires not only specific adult behaviors but also adaptations of development and emergence of the aquatic larvae. Here we examine the phenology of this cycle of development over a longer period and greater geographic range than heretofore. Its implications for genetic interchange between early and late emerging adults, and the light it sheds on the evolution of migration in *A*. *junius* are the foci of this paper.

## Materials and methods

The following describes four separate studies of the emergence patterns of *Anax junius* in the eastern half of the United States. Each was performed independently using somewhat different techniques, but together they provide both temporal and spatial detail and present data from a wide enough geographic area to consider how variation in climate and habitat at a continental scale can influence the evolution of migration in *A*. *junius*. In each study, exuviae were collected by careful inspection of potential emergence sites around the periphery of one or more ponds during the emergence season. In two of them, larvae were also collected from approximately April to October, using standard collection techniques for aquatic insects. Living dragonfly larvae were returned to the sites from which they were collected. No vertebrate animals or human subjects were involved in these studies, nor were any organisms that are protected or listed as warranting protection by any organization or government body.

Work at Patuxent National Research Refuge was conducted under permit NSR-CT-0303 (Migration Behavior of the Common Green Darner Dragonfly) to M. L. May. Studies at KHMO were carried out by J. and S. Gregoire on their own private property. Annual Rhode Island Department of Environmental Management permits for sampling at State Parks, Forests, Wildlife Management Areas were obtained by M. Aliberti Lubertazzi for work in Rhode Island; she received verbal approval to sample at other sites, including a variety of publicly-owned, privately-owned, and NGO-owned sites. Work by J. H. Matthews at the study pond at Austin, TX, which was located on the Brackenridge Field Laboratory campus of the University of Texas, was conducted with the permission of the University.

### Patuxent National Research Refuge (PNRR), Laurel, MD, USA

The study was conducted at PNRR, (ca. 39.04°N, 70.08°E), primarily at a pond complex known locally as Patuxent Marsh, an abandoned borrow pit divided by an incomplete dike into two sections. At its maximum the NE section (hereafter PM1) was roughly 2500 m^2^ and little more than 1 m deep, the SW section (PM2) about 3000 m^2^ and probably about 2 m deep; at high water the sections were broadly confluent at the S end of the dike but often became completely separated at low water in late summer. The pond was in young, mixed hardwood forest with a gravel road on the east side. Emergent vegetation in the NE section was a mixture of grasses, sedges, and a few woody shrubs while the SW section also had extensive *Typha*. The only fish noted before 2004 were Eastern Mudminnow (*Umbria pygmaea*), which are mostly bottom feeders on small Crustacea and Diptera larvae [32] but will feed on dragonfly larvae in aquarium experiments [33]. A few very small *Lepomis* sp. were found in 2004. Adult *A*. *junius*, including ovipositing females, were present in variable numbers throughout each year of the study from at least early May through September. This pond also supported a population of *Anax longipes*.

Larvae were sampled at irregular intervals between April and November from 1999–2004 using a dip-net with ~ 4x4 mm mesh. Efforts were focused on areas with submerged or emergent vegetation, since *Anax* larvae spend much of their time clambering in this habitat [34, 35]. For each larva maximum head width across the eyes was measured to 0.1 mm using a vernier caliper and body length (anterior margin of labrum to tip of epiproct) to 0.5 mm using a millimeter ruler; larvae were then returned to near their collection location. Head width was less subject to error and was used in plots of larval size vs. time. Exuviae were collected at 2–3 week intervals in 2004 and 2005 by examining all accessible emergent vegetation as well sticks, logs and tree branches that extended into the water around the margins of both pond sections. Aliberti Lubertazzi and Ginsberg [36] found that 50–60% of odonate exuviae are lost from plant stems within 3 weeks of emergence. Logistical considerations prevented more frequent collection at PNRR (and in Rhode Island, see below), so we clearly counted only a fraction of the *A*. *junius* that emerged, but since collections were at fairly uniform intervals, and the timing of emergence was consistent with larval development, the basic pattern of emergence should be reflected in our data, albeit with less detail than at KHMO (see below).

A much larger (ca. 24 ha) pond at PNRR, Millrace Pond, was also investigated, although sampling was less frequent. In 2004 only, exuviae were sampled on each day that sampling was conducted at Patuxent Marsh, along an irregular transect including about 50 m of the immediate shoreline and also areas of emergent vegetation and snags up to 20 m from shore. This pond had extensive beaver workings, with many floating and submerged snags and areas of dense herbaceous and woody vegetation, forming a physically very complex subsurface environment. Small sunfish (*Lepomis* spp.) were abundant, as were adult *A*. *junius* flying over the pond.

### Kestrel Haven Avian Migration Observatory (KHMO), Burdett, NY, USA

The study pond (42.4438°N, 76.7578°W), constructed in 1999, is fed via a drain tile from an artesian spring, has a maximum depth of ca. 5.5 m, a surface area of roughly 1400 m^2^ and is relatively steep sided; it is normally stratified in summer. Marginal emergent vegetation consists of *Typha* and sedges, with extensive submerged *Chara* covering most of the bottom. The surroundings are mixed forest, old field, and hedgerow with a short length of turf. Fathead Minnows (*Pimephales promelas*), which are primarily omnivorous benthic filter feeders that also take zooplankton and very small insect larvae [37] are the only fish present; they are unlikely to be important predators of *A*. *junius* larvae.

Larvae were collected using a standard bottom trawl net in 2004 only, and head width was measured as above. Exuviae were collected daily from 2004–2013. Each morning during emergence season we conducted a thorough search of all possible emergence structures by wading along the entire pond edge and closely examining cattail (the preferred substrate), reed, rush, grasses and the dock. We removed all exuviae and also looked for floating exuviae that had fallen off the emergence substrate and incompletely emerged individuals.

For each year of emergence data we made a rough test of bimodality by fitting a fourth-order polynomial to the number of exuviae found as a function of day of year. If the fourth order term was significant, the pattern was considered to be bimodal.

### Rhode Island, USA

The study area, which included ponds throughout the state of Rhode Island (RI), lay between approximately 41.2–42.0°N and 71.1–71.8°W. *Anax junius* exuviae were counted at a subset of the ponds surveyed each year. Three were sampled in 2004–2006, 15 were sampled in 2004–2005 only, and 11 were sampled only in 2006. The 29 ponds where *A*. *junius* exuviae were collected in at least one year were 0.05–1.78 ha in area; (mean = 0.36 ha, SE = 0.09 ha; median = 0.14 ha), most held water throughout the study (5 were dry or nearly dry during part of Aug.-Sept. 2005), and 13/29 lacked fish populations. Their locations ranged from 37.51 m to 29.41 km from the maritime coast (mean = 8389.13 m, SE = 1487.66 m; median = 7570.06 m). Sampling usually occurred at intervals of 2–4 weeks at each pond [38]. Because sampling around the entire perimeter of some ponds was not possible, results are given as exuviae per hour of effort rather than as exuviae per pond.

### Austin, TX, USA

Emergence in the field was measured quantitatively by making exhaustive collections of exuviae left on emergence supports at a small semi-permanent pond on the campus of the University of Texas during portions of the years 2003–2005. The study pond was one of several built at the Brackenridge Field Laboratory, all of which can be filled via pumped groundwater but during the study period were filled by precipitation and runoff. As a result, the pond had shifted to an ephemeral state. The study pond is shallow (nor more than 0.5 m maximum depth), square, with a smooth and uniform clay bottom, about 1 hectare in area; no resident fish species were observed. Matthews had observed the pond to become completely dry during summer in years before and after the study period. Dense mats of submerged aquatic plants formed when the pond was full, including *Najas guadalupensis* and *Elodea canadensis*. Following Trottier’s collection guidance, some 10 m of 1.5 m high 4x4 mm netting ringed the edges of the pond, about 0.75 m from the shore, and supported by posts every three meters. Inspection of the netting occurred every one to three days during emergence season and about once weekly during the non-emergence season. Collections over the three years encompassed January to December, although the maximum period in any one year (2004) was March–December.

### Analysis of public records

In order to construct a broad picture of adult activity as a rough proxy for emergence phenology throughout the United States, we acquired data from Odonata Central (OC) [39], the Florida State Collection of Arthropods (FSCA), and records supplied by D. R. Paulson (DRP) on confirmed sightings and collections of adult *Anax junius* from states in which the combined number of records exceeded 50, except that the states along the northern Gulf of Mexico (AL, MS, LA), which had very similar patterns of occurrence, were combined to obtain a full sample. The number of records each month and the sex of each specimen was noted. Data from OC and DRP usually also included GPS location, but from FSCA only the county where collected was recorded. We assume that these records represent a random sample of occurrence, although bias could occur due, e.g., to spatial or temporal restrictions of especially prolific collectors. Because each state total originates from records accumulated over a number of years and a large area, including migrants from other regions as well as individuals that emerged locally, they give an imprecise picture of phenological events. Nevertheless, such data are currently the only source of information at a country-wide scale.

## Results

### Patuxent Research Refuge

**Fig 1:**
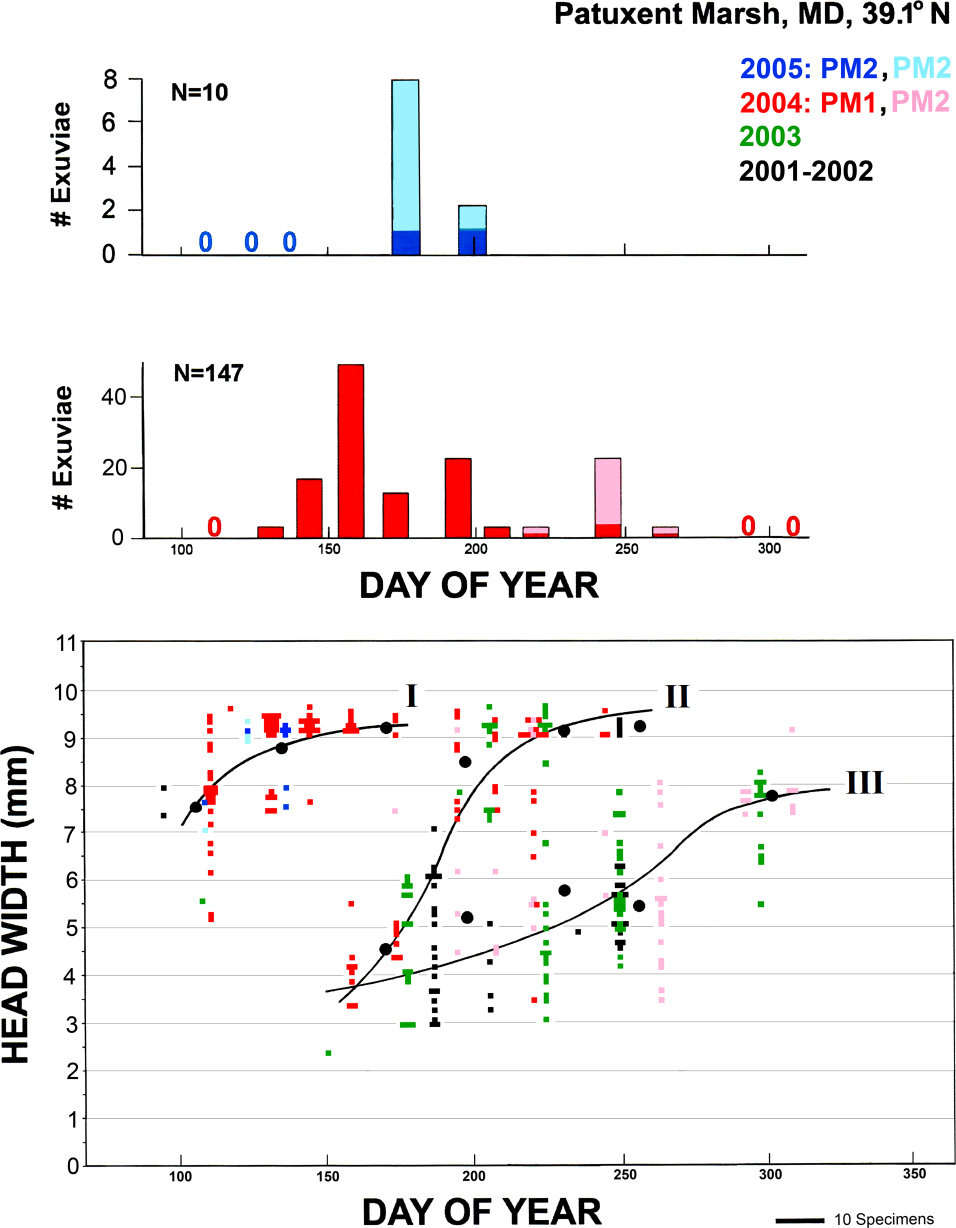
Larval growth and emergence of *Anax junius* at the “Patuxent Marsh” ponds 1 (PM1) and 2 (PM2) at PNRR, Laurel, MD. The lower graph shows the number of larvae (shown by the width of each bar) of a given head width on the collection day (y-axis); different colors indicate data for different years, 2001–2005 (underlying data in S1 Table). Black dots and curved lines represent the size, averaged for each calendar month, of groups of larvae (separated by eye) thought to comprise primarily individuals that: (I) had overwintered in Patuxent Marsh; (II) were offspring of migrants, from eggs deposited in spring; and (III) were offspring of adults from group I and perhaps some late group II larvae. The upper graphs show the number of exuviae collected on each indicated date in 2004 and 2005 (underlying data in S2 Table); darker colors indicate exuviae collected from PM1, lighter colors those from PM2; zeros along the x-axis indicate sampling days when exuviae were sought but none were found. To facilitate comparison of patterns of emergence, bars representing the maximum number of exuviae collected on a single day of each year are set to the same height; consequently, the scale on the y-axis varies markedly between years.

Development of larvae at PNRR, although inferred from data taken at varying intervals from 1999 to 2004, appears to have followed a reasonably stable pattern. Most larvae were collected in the shallower NE pond sections, but no difference in size distribution were evident between the two sections; mean head widths did not differ between PM1 and PM2 during the periods when both sections were sampled, July-September 2004 and May 2005 (t-test, p>0.2 in both cases). At least two and possibly three trajectories of growth (Fig 1) can be discerned. One group of larvae (I in Fig 1), which must have overwintered, was half- to fully grown by mid-April and emerged for the most part by the end of May. From early June through mid-August smaller larvae were also collected; larvae with head width <3 mm were rarely collected, although the first six instars are smaller than this [40], probably because of low netting efficiency and detectability. The earlier individuals of this size develop rapidly and emerge from July (probably overlapping with late-emerging individuals from the group i until mid-October (II in Fig 1); the later small larvae seem to have developed more slowly (III in Fig 1). These probably represent the offspring of adults that emerged in May and that, like their parents, would overwinter at the Patuxent Marsh ponds. Note that the size distribution of the larvae collected in late October and early November is very similar to that of larvae collected in April(ca. 5.5–8 mm head width), suggesting that the former would overwinter without further growth until spring.

Emergence was quantified only in 2004 and part of 2005, from May to October. During the first part of 2004, collecting was confined to PM1, and collections were made by searching the entire perimeter as well as emergent stems throughout the pond. By late July, however, that portion of the pond became quite shallow and warm, with red-brown flocculence developing in the southeastern ~1/3, and both larvae and exuviae became very scarce. Probably because of its greater depth, PM2 was slightly cooler and lacked flocculence. Initially, exuviae were few in that section as well, but thereafter both exuviae and larvae were more readily collected, and from early September onward, 25 of 29 larvae were collected in the PM2, again around the entire perimeter and on as much emergent vegetation as was accessible. Concentrated collecting in PM2 began in early July when signs of deterioration were first noted in PM1; during July and August, 13 of 41 larvae were taken in the latter (for the difference between July and Aug. vs. Sept. and later, χ^2^ = 10.9, p < 0.05). Although larvae were not collected in 2005 and exuviae could not be collected after 18 July, it is apparent that a severe population decline occurred in PM1, but not in PM2, of the pond in midsummer of 2004, and this may have been reflected in low emergence in early 2005.

**Fig 2:**
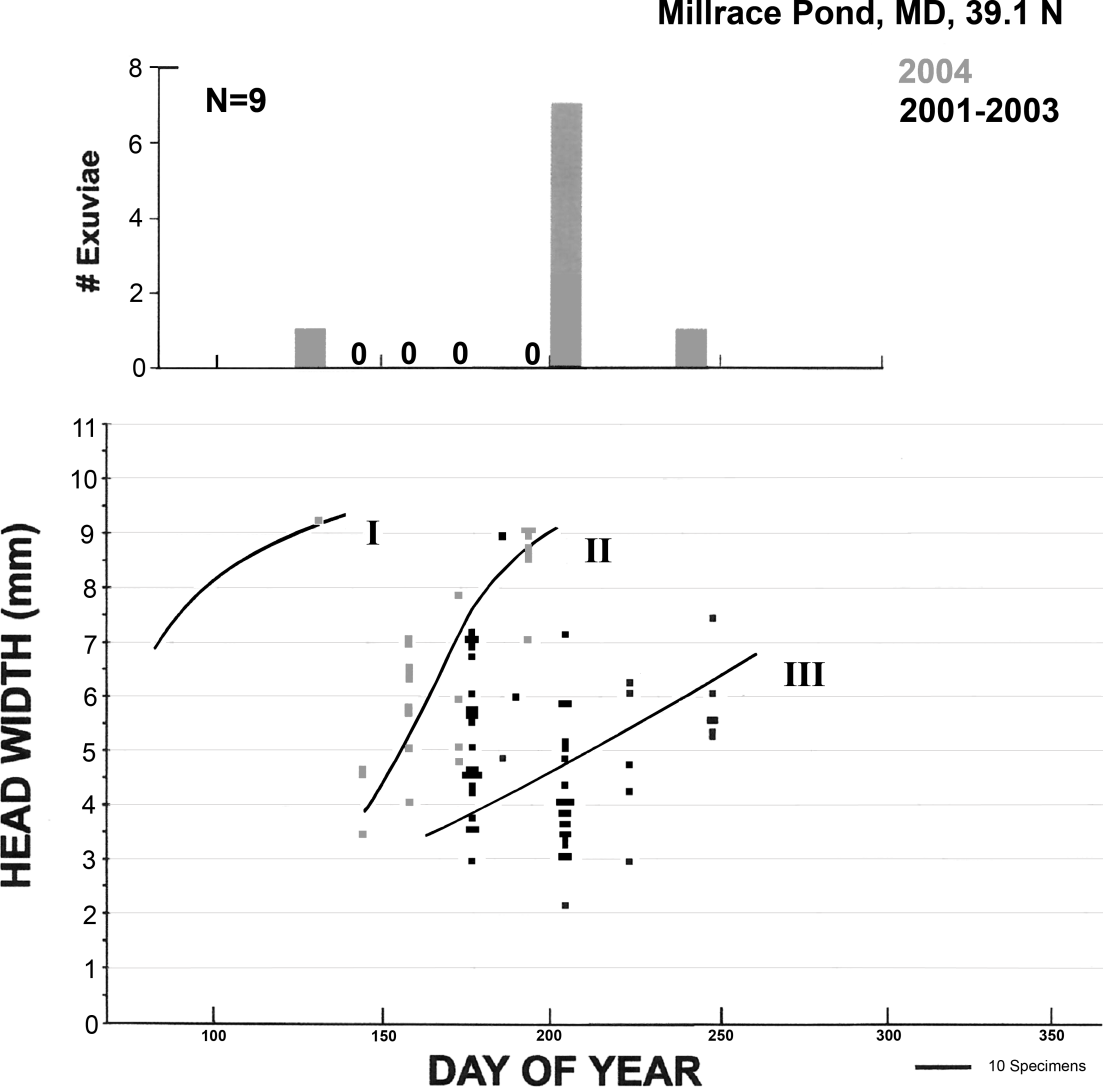
Larval growth and emergence of *Anax junius* at Millrace Pond at PNRR. Plots as in Fig 1 (underlying data in S1 Table) except that collection periods are shown in shades of grey, with 2001–2003 data lumped. Hypothesized larval growth trajectories as in Fig 1; note that very few larvae apparently overwintered successfully. Exuviae (upper graph; underlying data in S2 Table) were collected only in 2004; the bar representing the maximum collected on a single day is set to the same height as the maximum in Fig 1.

Results from Millrace Pond (Fig 2) are not precisely comparable to those from Patuxent Marsh because only a small fraction of the former was surveyed, and movement, dip-netting, and detection of exuviae were more difficult. No signs of habitat deterioration were noted, but observed emergence was scant, with a distinct but small burst of emergence in late July. Adult males were commonly seen patrolling throughout the summer.

### Kestrel Haven

**Fig 3:**
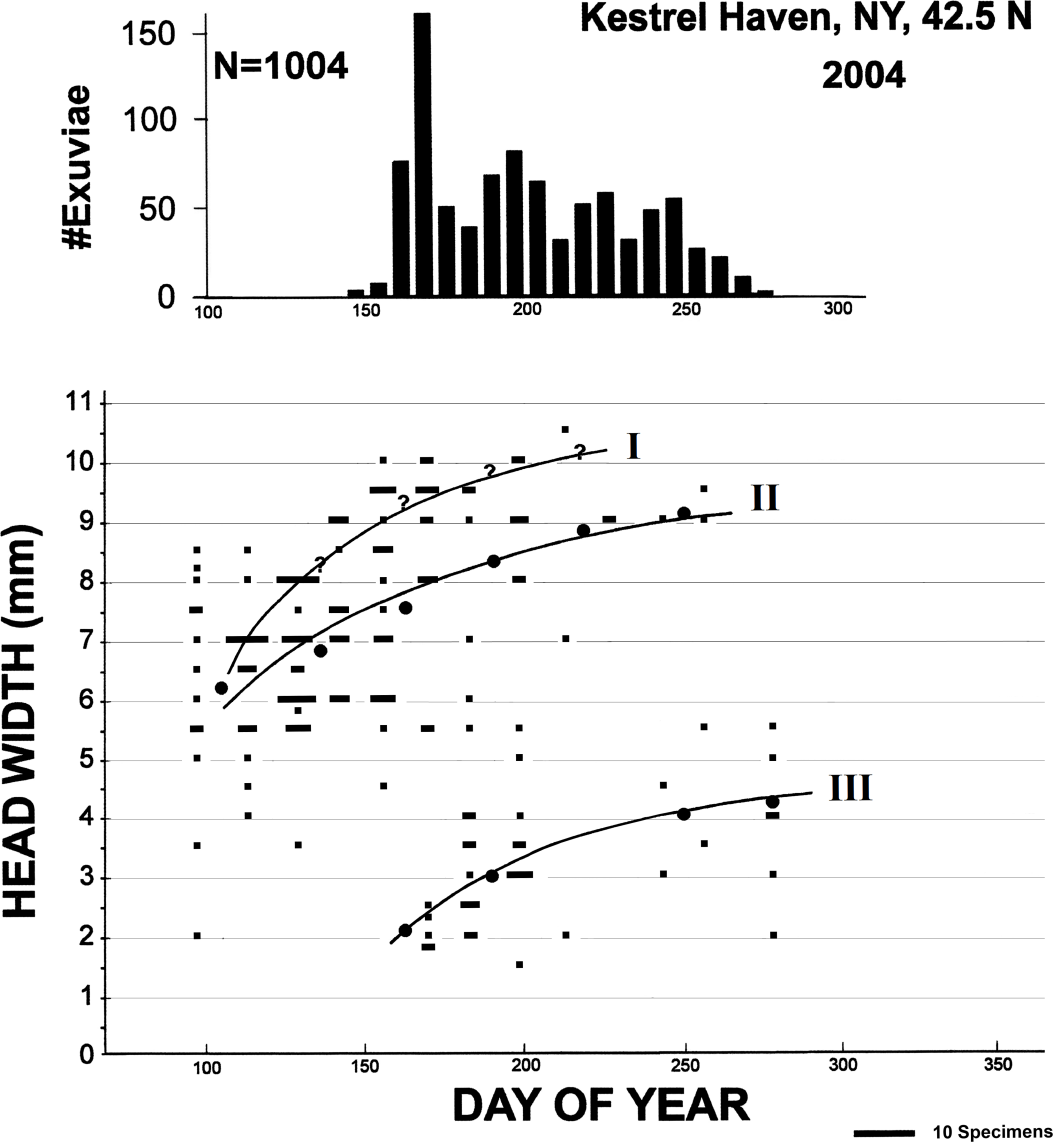
Larval growth and emergence of *Anax junius* at the study pond at KHMO in 2004. Larvae were collected weekly. Lower plot as in Fig 1, upper plot as in Fig 1 except that the number of exuviae collected are summed and plotted over one week intervals. The bar representing the maximum collected during one week is set to the same height as the maxima in Fig 1. Underlying data in S3 Table.

The pattern of larval development at KHMO in 2004 (Fig 3) was quite similar to that observed at PNRR. Larvae with head widths of ca. 9.5 mm or more in early June were probably very close to emergence and contributed to the sharp first peak of emergence in mid- to late June. Smaller numbers of last instar larvae continued until at least mid-September and evidently contributed to the extended emergence until October. The absence of larvae with head width > 6 mm by October contrasts with PNRR and is unexpected given that data from April at both sites suggest that larvae passed the previous winter in larger instars.

**Fig 4:**
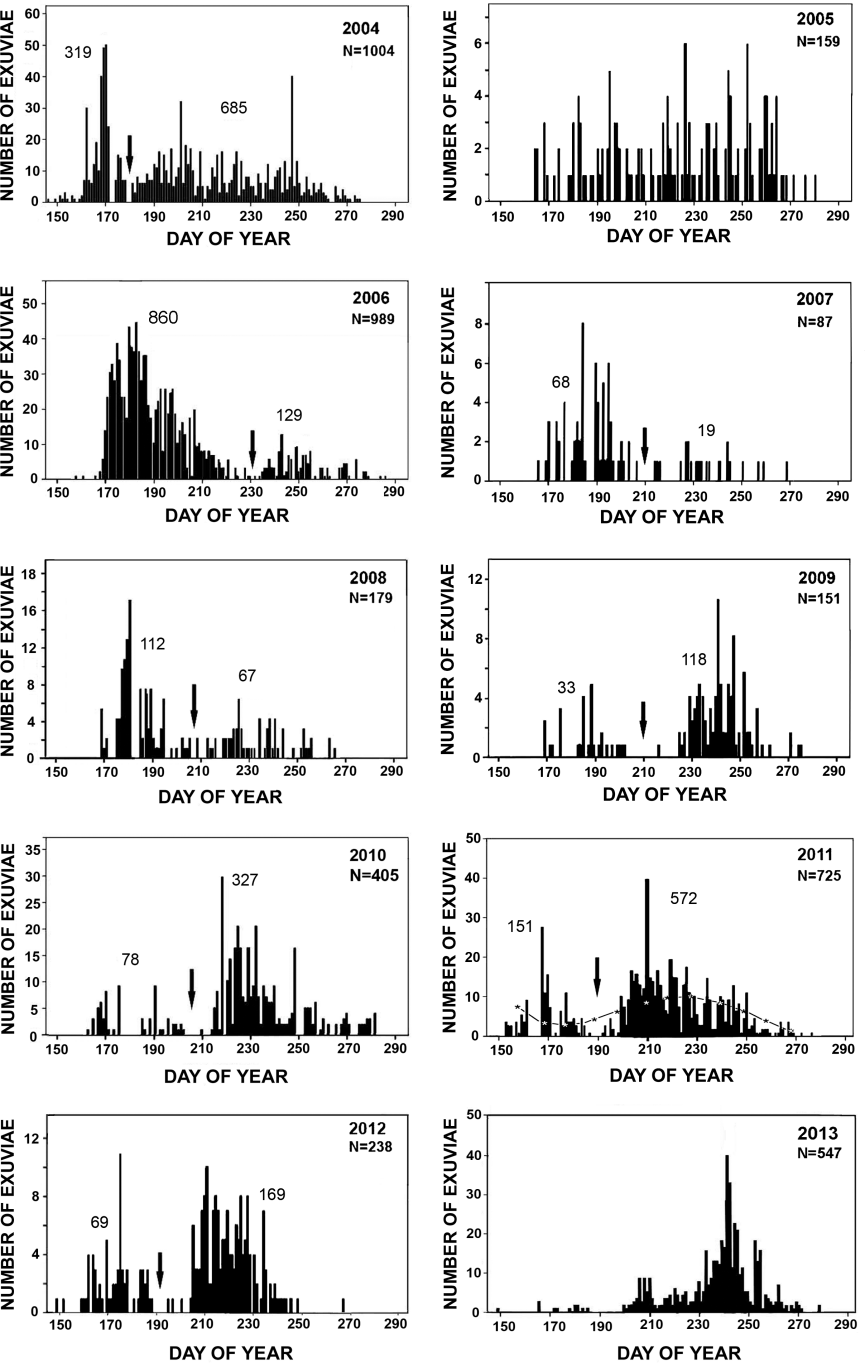
Number of *Anax junius* exuviae collected throughout the entire emergence periods of 2004–2013 at the study pond at KHMO, Burdett, NY. Exuviae are plotted by day of year; each vertical line indicates one day’s collection. The bars representing the maximum collected on a single day in each year is set to the same height as the maxima in Fig 1, so that the vertical scale of the graphs varies among years. Downward pointing arrows indicate the estimated minimum between early and late seasonal peaks for years in which a bimodal pattern of emergence was confirmed; total number of exuviae for the year (N) is at upper right, beneath year, totals for early and late episodes of emergence appear above the corresponding peak; narrow curved line and asterisks on plot for 2011 shows an example of fitted 4^th^ order polynomial. Underlying data in S4 Table.

Full emergence results from KHMO represent the most complete data set for any odonate species known to us, with 10 consecutive years of almost daily collections of *A*. *junius* exuviae from a single pond (Fig 4). Variability in the temporal pattern and the magnitude of emergence are striking. In 2005 no clear emergence peaks were evident, and only 159 exuviae were collected in total. In 2006, 989 emerged, with very large peak in early June (ca. day 150), a distinct but much smaller peak in early September (ca. day 250), but very low numbers around the July-Aug boundary. In 2008 the pattern was similar to 2006 but the peaks were less distinct and were both slightly earlier, with total annual emergence only 179; in 2011 two clear peaks occurred again, but both were earlier still and the later peak (ca. day 210, early Aug) was clearly larger, with 725 emerging overall. Other years show similar variation, always with some successful emergence, but annual totals ranged from 87 (2007) to 1004 (2004). Thus, even in this relatively stable pond, annual emergence varied over more than an order of magnitude. Peak emergence met our criterion for bimodality except in 2005 and 2013, but there was never a complete hiatus in emergence of longer than 12 days, and the major peak of emergence varied from ca. day 170 (2004) to almost day 250 (2009, 2013). This is consistent with the patterns of larval size both at PNRR (Fig 1) and in 2004 at KHMO (Fig 3).

We investigated whether local climatic conditions might have affected emergence success or timing at this site (Table 1). Surprisingly, mean air temperature had no effect. Total emergence and the numbers emerging during the first peak (where applicable) were positively correlated with rainfall during the months of emergence (April-October) of the current year. Neither precipitation nor temperature had significant effects on the relative timing of the peaks, the size of the second peak (with one exception), or the ratio of the sizes of the early and late emerging groups. It is possible that conditions during the previous year may have affected emergence, especially in overwintering larvae, but this could not be analyzed independently because of the relatively small year to year changes in weather and the large overlap of the range of years (i.e., 2003–2012 vs 2004–2013).

**Table 1:** Correlation of emergence characteristics with rainfall during various periods of the year at KHMO.

TIME INTERVAL	Jan- Mar	Mar-May	May-Jul	Jun-Aug	Jul-Sept	May-Sept	Apr-Oct	Annual
**EXV**^**a**^	-0.226	+0.561	**+0.718**	+0.555	**+0.856**	**+0.926**	**+0.902**	**+0.678**
	0.558	0.116	**0.0290**	0.121	**0.0033**	**0.0003**	**0.0009**	**0.0447**
**PK1**^**b**^	-0.018	+0.017	**+0.900**	**+0.887**	**+0.907**	**+0.926**	**+0.843**	**+0.727**
	0.967	0.967	**0.0023**	**0.0033**	**0.0019**	**0.0010**	**0.0085**	**0.0412**
**PK2**^**c**^	-0.245	**+0.817**	+0.017	+0.207	+0.338	+0.397	+0.439	+0.266
	0.559	**0.0134**	0.910	0.623	0.412	0.329	0.277	0.524
**DIV**^**d**^	+0.324	-0.501	+0.497	+0.670	+0.232	+0.266	+0.296	+0.294
	0.483	0.206	0.210	0.0692	0.550	0.525	0.477	0.480
**RPK**^**e**^	-0.397	-0.346	+0.496	+0.597	+0.329	+0.370	+0.442	+0.0741
	0.330	0.401	0.211	0.118	0.428	0.368	0.273	0.862

Upper number in each cell is Pearson correlation coefficient, lower number is probability of obtaining a higher correlation by chance; significant correlations (p<0.05) are in bold font. Underlying data in S11 Table

^a^Total exuviae for the year.

^b^Exuviae in 1^st^ emergence peak.

^c^Exuviae in 2^nd^ emergence peak.

^d^Day at which peak 1 was divided from peak 2.

^e^Ratio of number of exuviae in peak 1 to number in peak 2. Note that b-e do not include years for which no 2^nd^ peak was apparent (2005, 2013).

### Rhode Island

**Fig 5:**
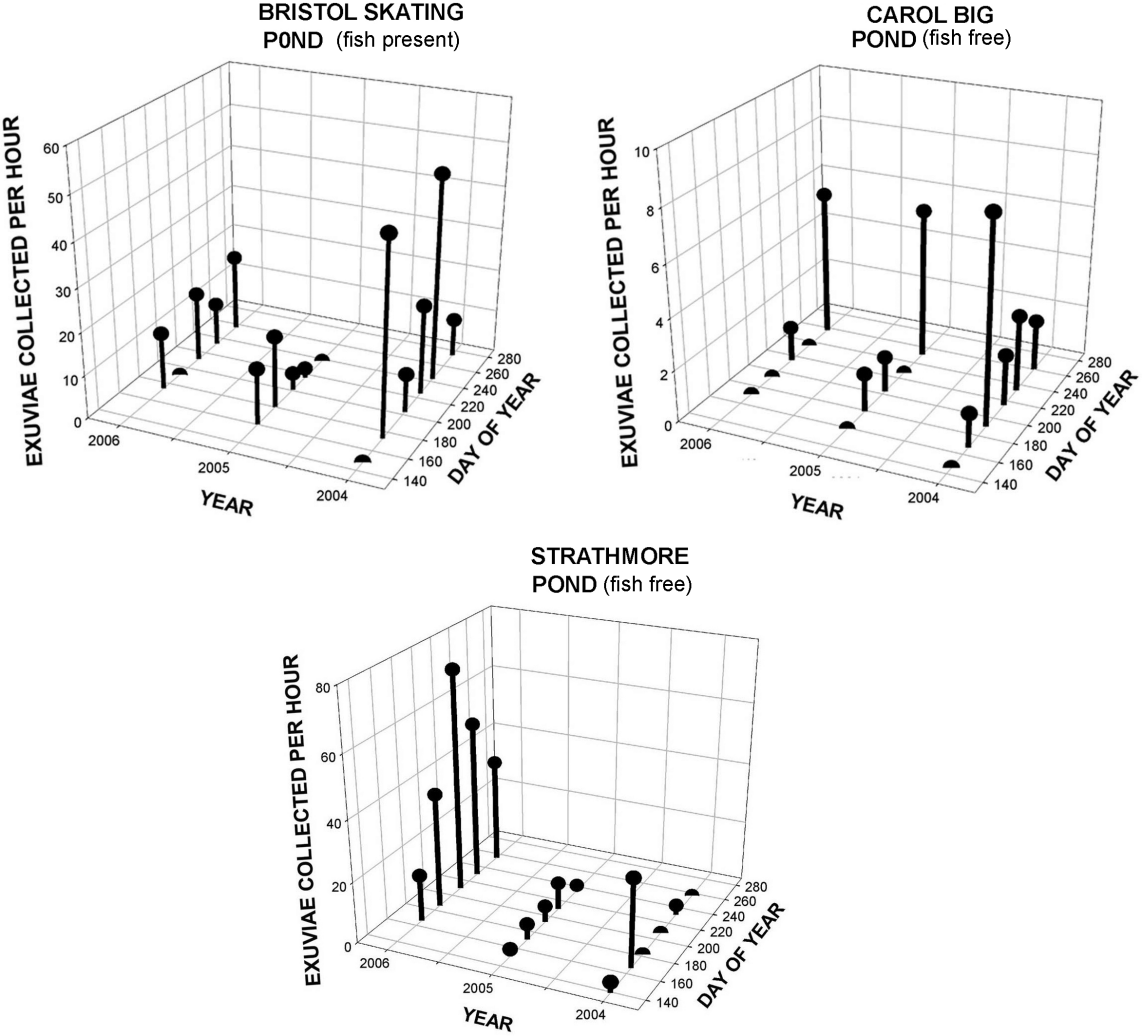
Numbers of *Anax junius* exuviae collected per hour of effort vs year and day of year at three Rhode Island ponds where collections were made for three years. Only Bristol Skating Pond supported fish; Bristol Skating Pond and Carol Big Pond were dry in August and September 2005, respectively, and Strathmore may have dried in September of that year. Underlying data in S5 Table.

**Fig 6:**
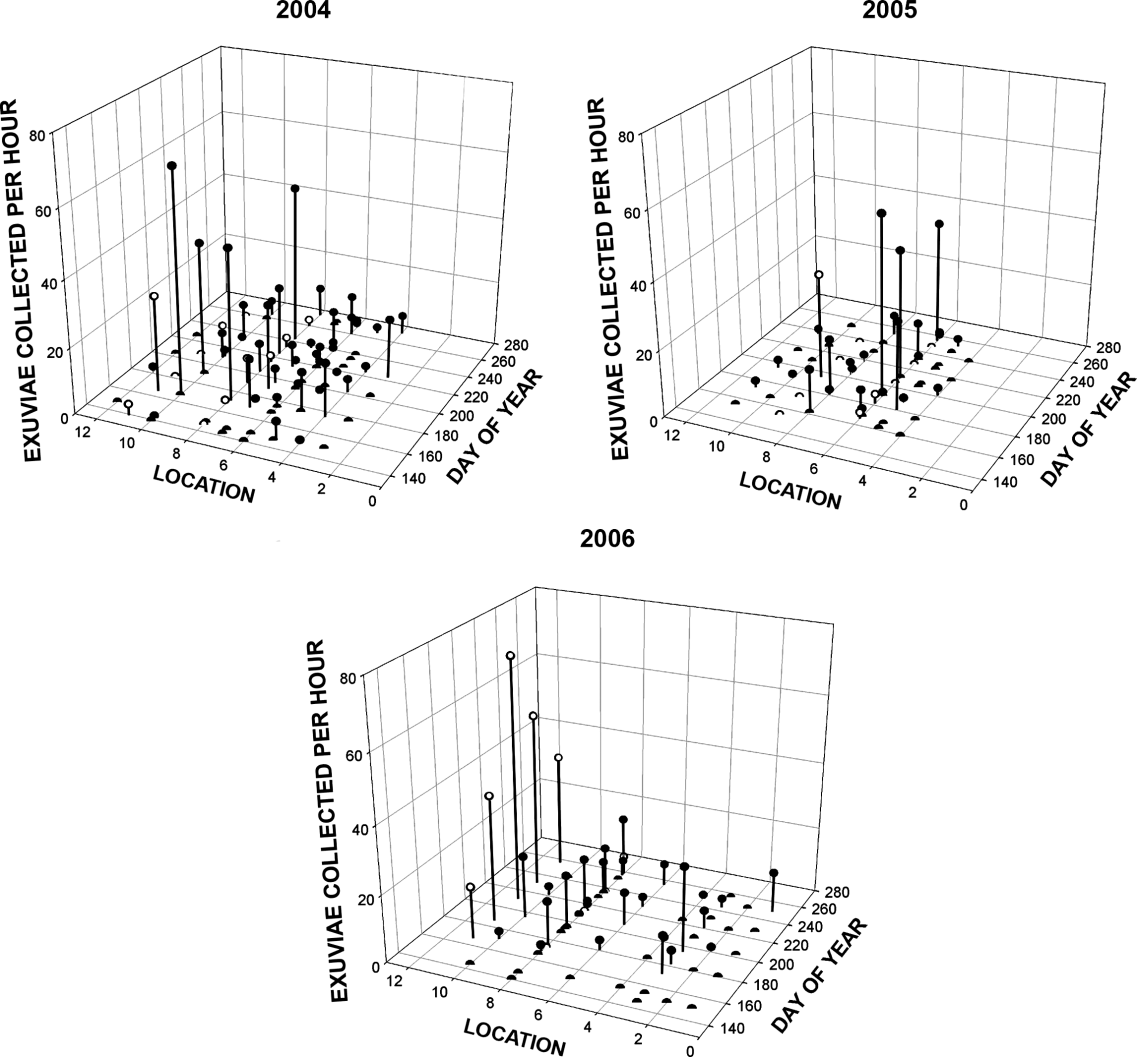
Numbers of *Anax junius* exuviae collected vs day of year and location (see text) for all 29 Rhode Island ponds surveyed in each of three years. In addition to those listed in Fig 5 and S2 Fig, two ponds, Amtrak and Nbground, at locations 9 and 6 respectively on the 2006 plot, were fish free (open circles). Underlying data in S5, S6 and S7 Tables.

Our third extensive emergence dataset was temporally less fine-grained that that from KHMO, but it included emergence sites spread over about 3000 km^2^. Despite relatively infrequent sampling, apparent unimodal and bimodal emergence patterns occurred (Figs 5, 6 and S1); samples at any one pond, however, were too few to permit a statistical test of bimodality or to clearly identify times of maximum emergence. From Fig 5 it is evident that all three ponds that were sampled for three consecutive years showed substantial variation in total numbers emerging in different years, as well as in seasonal patterns of emergence, although we did not formally test the latter owing to relatively sparse and inconstant intersample intervals. Similar patterns occurred in many of the ponds sampled for two years (S1 Fig). Fig 6 shows emergence patterns in each of the three study years from all ponds sampled; the x-axis is a rough measure of location, the displacement due southeastward from a SW–NE line running through the NW corner of the state. Overall emergence success was significantly lower in 2005 (2004–5.56±1.21 SE, 2005–4.28±1.16, 2006–6.091.52) than in 2004 and 2006, and 2005 was also the year with the lowest summer rainfall at the nearby Theo Francis Green Airport in Providence, RI (2004–68.7 cm; 2005–48.3 cm; 2006–72.8 cm). Five of the ponds were dry or nearly dry during part of August and September of 2005, although it is evident from Fig 6 that emergence was reduced even earlier. Effects of year were significant (p = 0.001), as was pond identity (p<0.0001), but not day of year, (p = 0.637). Location had a significant effect overall (p<0.0001 controlling for day, p<0.0002 irrespective of day) and in each year individually (p<0.05 in 2004. P<0.0001 in 2005 and 2006); nevertheless, the actual locations with greatest emergence success varied markedly across years (Fig 6). For the entire study, 9.86 exuviae were collected per hour in fish-free ponds vs. 3.24 in ponds with fish; emergence was marginally lower in ponds with fish than in those without in 2004 (p = 0.083) and highly significantly so in 2005 and 2006 (p = 0.0035 and 0.0028, respectively). In some years a marginally significant (p< = 0.10) negative relationship was found between exuviae collected per unit effort and pH and a positive relationship with forest cover adjacent (within 100 m) to the pond [38].

### Southern United States

**Fig 7:**
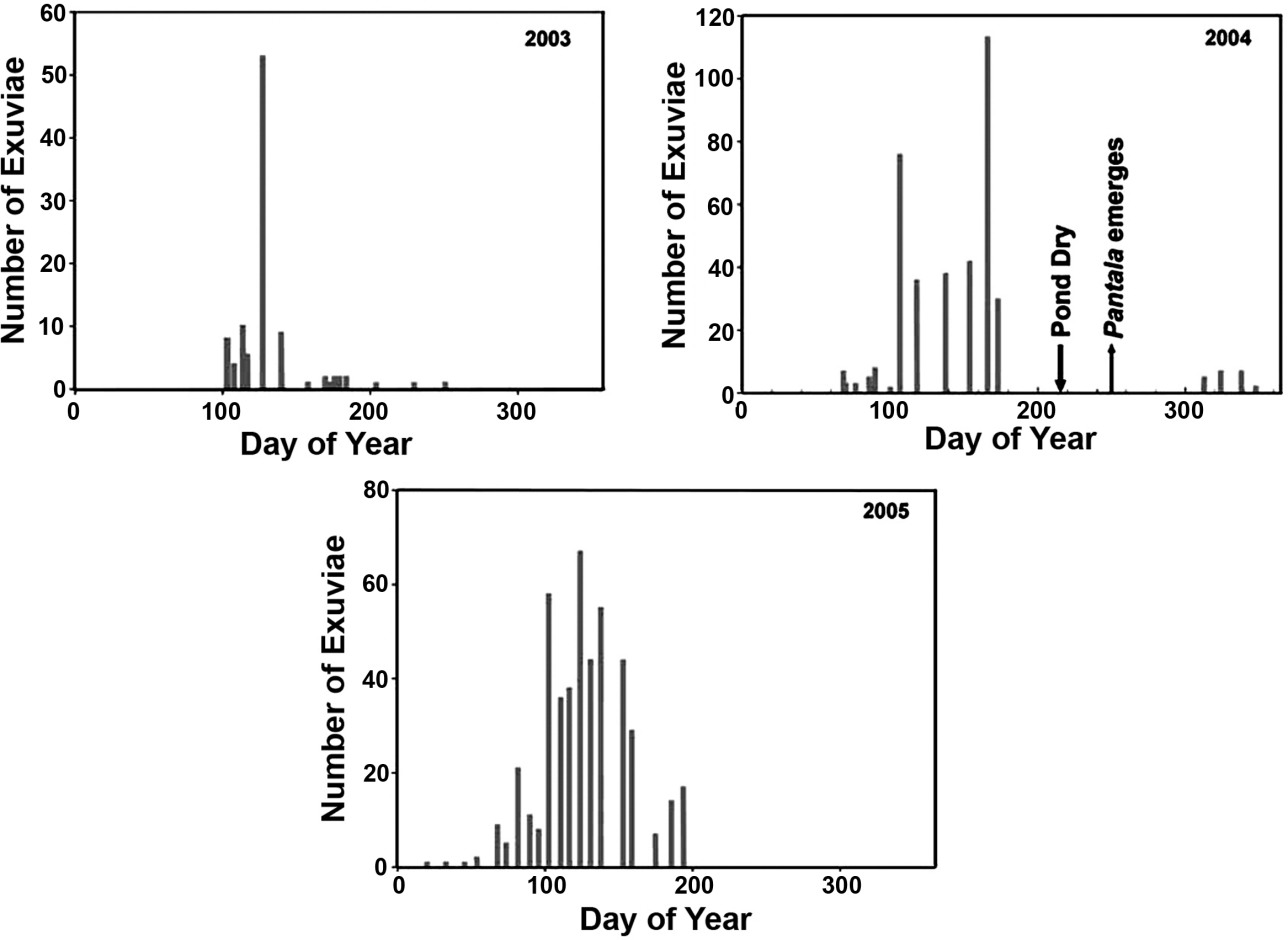
Numbers of *Anax junius* exuviae collected throughout the varying portions of the emergence periods in 2003–2005 at a single pond in Austin, TX; each vertical line indicates one day’s collection. Note that the scale of the y-axis varies among plots. During 2003 and 2005, collections were ended at about day 200 and day 250, respectively. Downward pointing arrow indicates the date on which the pond became almost completely dry and anoxic in 2004; upward pointing arrow indicates emergence, after partial refilling, of *Pantala flavescens*. Underlying data in S8 Table.

To our knowledge comprehensive data on larval growth or emergence are not available from the southern portion of the range of *A*. *junius*, south of about 39^o^ N. Observations by JHM at Austin, TX, with somewhat irregular sampling (Fig 7), shows a clear peak in early to mid-May in 2003 and 2005; collecting ended in late summer and fall (associated with extended dry periods, causing the pond to become completely desiccated). In 2004, most emergence was in early May to mid-June. Drought in July led to low water, high water temperature, and O_2_ depletion, and no emergence occurred in July or August; the pond was completely dry for an uncertain period ending near the beginning of August. *Pantala flavescens*, a migratory libellulid dragonfly known for its very rapid larval development, emerged by early September and a few *Anax* emerged from October to early December, presumably having hatched after the pond refilled, since *A*. *junius* larvae are not known to be drought resistant.

**Fig 8:**
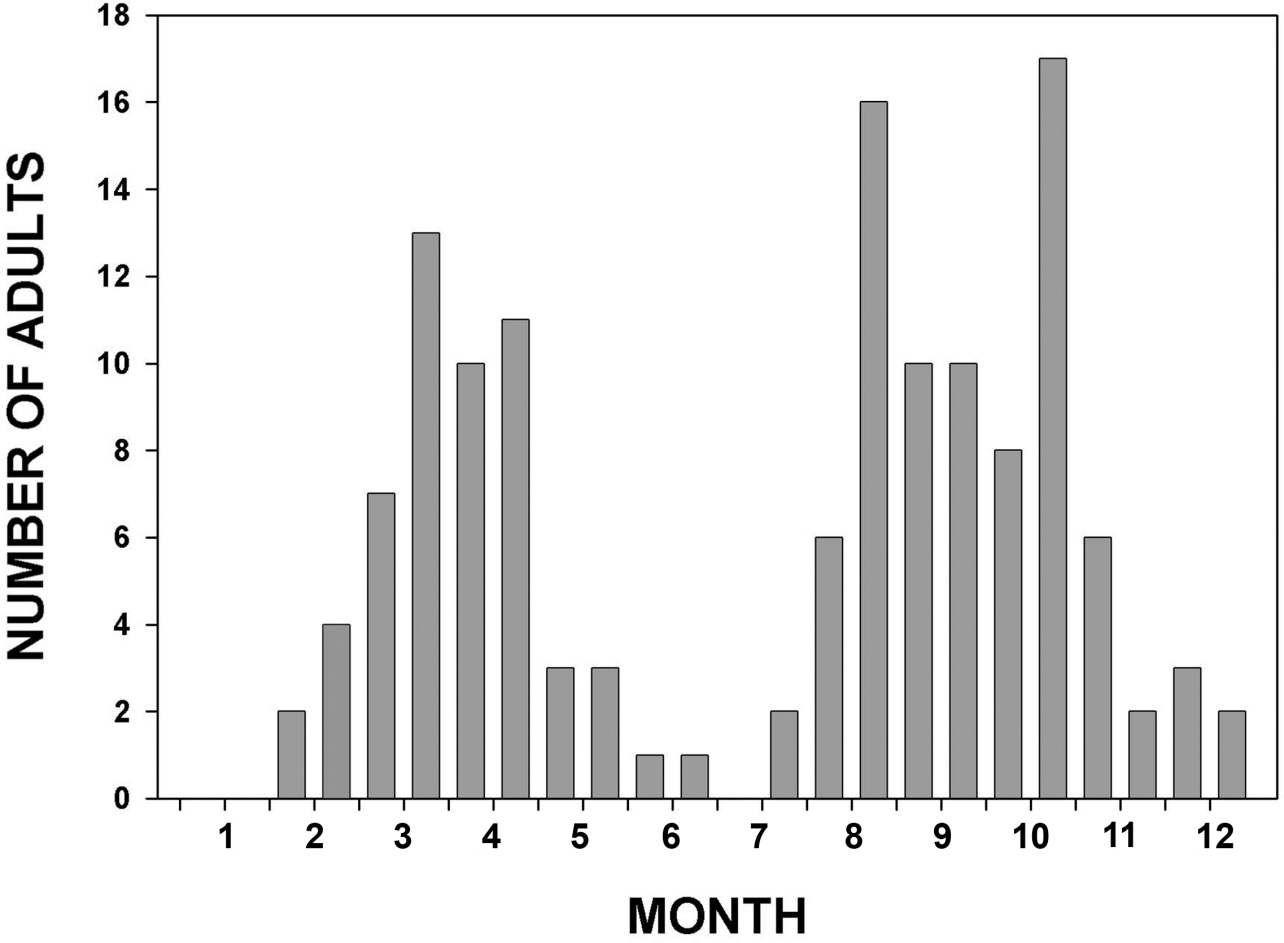
Number of adult specimens of *Anax junius* in the Florida State Collection of Arthropods collected from Jan. to Dec. in peninsula Florida south of 29°N. Vertical bars represent the total number per two week interval. Underlying data in S9 Table.

In the early 1960’s in southern Florida Paulson [41] collected adults and exuviae as part of a general survey of south Florida Odonata at ca. 24.5–27.5°N. He recorded a sharp emergence peak, based on exuviae, in March but collected relatively few adults; the major peak of adult activity occurred from August to October and comprised mostly fully mature individuals, with a small emergence peak in October. In 2011, we examined all adult *A*. *junius* in the Florida State Collection of Arthropods that were collected in Florida south of ca. Daytona Beach (~29°N) (Fig 8). Assuming uniform collecting effort, this confirms two very distinct peaks of adult activity at about the times observed by Paulson, although the spring vs. fall disparity is not as great. Moreover, the great majority of individuals in the early peak were sexually immature (based on color and cuticle stiffness) when collected, while nearly all in the late peak were mature. Data from OC and FSCA for the entire state showed a generally similar pattern, with a smaller adult peak in spring, a larger one in late summer and fall, and almost no adults from May-July.

## Discussion

The genus *Anax* is probably of tropical origin [13], but *A*. *junius* and several other species have adapted to permanent occupation of temperate habitats by passing the winter in larval diapause. In this they resemble many other North Temperate Zone species that exhibit obligate or facultative larval diapause [13] and are thus able to overwinter in cold regions. Unlike most odonate species, however, many adult *Anax* migrate long distances southward in autumn and lay at least some of their eggs in regions where diapause is probably not required (average temperature is mild even in winter, > = 15 ^o^C, on much of peninsula Florida and the south Texas and Mexican Gulf Coast), although the effects of photoperiod at these latitudes are unknown. The question then arises, why employ this dual strategy, which seemingly complicates life history adaptations and entails multiple sources of risk?

Results from both PNRR and KHMO show that early- and late-emerging adults at these latitudes do not form clear-cut cohorts and in most years must certainly have the opportunity to interbreed. Thus the difference between “residents” and “migrants” is unlikely to be underpinned primarily by genetic differences, as previously suggested [29, 30]. Based on those population genetic studies and on evidence from stable isotope composition [15], May and Matthews [16] and May [19] suggested that an important advantage of long-distance migration to *A*. *junius* is that it allows migrants to spread reproductive risk across multiple, widely scattered water bodies, ameliorating threats from predation, intra- and inter-specific competition, and drought. Schenk, et al. [42] directly observed risk-spreading by oviposition in multiple ponds by the migratory dragonflies, *P*. *flavescens* and *Sympetum fonscolombii*, albeit on a smaller spatial scale than we suggest here.

### Choice of oviposition sites

Our results from Millrace Pond at PNRR make clear that *A*. *junius* will oviposit freely in waters inhabited by insectivorous centrarchid fish. Some larvae can survive in their presence if habitat structure is sufficiently complex [43], but such populations probably suffer substantial mortality and at Millrace Pond appear to have had very poor overwintering success. MLM has observed numerous ovipositing *A*. *junius* at Helmetta Pond in NJ (40.378 N, 74.427 W) in vegetation adjacent to multiple nests of *Lepomis* spp. Over many years of intermittent collecting of larvae at this locality, only one final instar *A*. *junius* larva was recovered. On the other hand, *A*. *junius* will also oviposit in very ephemeral pools that may persist only for weeks or at most 2–3 months, as long as some emergent vegetation for oviposition develops (May, pers. obs.). These incidental observations suggest to us that, like *Enallagma* spp. [44], *A*. *junius* cannot reliably detect the presences of predaceous fish in potential reproduction sites and also have limited ability to discriminate among sites on the basis of habitat stability, both of which must add to the uncertainty of successful reproduction at any given site.

### Variation in emergence success

Typical larval habitats commonly persist long enough to develop sufficient aquatic vegetation for oviposition but are usually ephemeral enough and/or isolated enough to be free of predaceous fish. Such habitats often are sufficiently stable to produce annual generations for several successive years, but most eventually become unsuitable through drought and its attendant abiotic stressors or by introduction of fish. Even in the absence of identified sources of mortality, emergence success can fluctuate sharply, as at KHMO. Emerging adults, therefore, have to balance risks of reproducing in their natal pond or region vs. those of undertaking long distance migration. Here we have tried to demonstrate the extent and nature of some of these risks by documenting variation in emergence success at several spatial and temporal scales.

We observed two instances in which study ponds or major portions of them became almost wholly unsuitable for development of *A*. *junius* larvae despite having supported substantial populations earlier the same summer: 1) at PNRR in 2004 the larval population of PM1 crashed during August, although water was still present, and it had not fully recovered as of July 2005; 2) the pond at Austin dried completely in 2004 but did produce some adult *Anax* in late fall after refilling. Clearly, from the second example, rapid recolonization and emergence is possible in the fall in warm regions. Recolonization during the same year must be much less likely further north because adult activity is prevented by cold, and larvae must reach a minimum critical size to enter diapause [13, 45].

Perhaps more surprising, however, is the extreme variability in emergence success in the relatively stable pond at KHMO. This pond never dried out, nor did its depth drop below the level at which marginal vegetation was readily available for oviposition and emergence, and water volume was clearly sufficient to prevent either complete freezing or warming to lethal levels The cause of the large fluctuations in emergence is not clear. Variation in numbers of larvae emerging could result from variation in recruitment via oviposition, but each year adults visited the pond in moderate numbers, and several nearby ponds also had substantial adult and emergent populations. Possibly the absence of small larvae in Fall 2004 indicates slow development and/or poor survival of the offspring of the early peak of adults and thus could provide a proximate explanation for the absence of a distinct early peak of emergence and for poor emergence overall in 2005. Larval sampling ended earlier at KHMO than at PNRR, however, so further growth might have occurred than we recorded. Moreover, the greater depth at KHMO may have buffered temperature change [46] so that development was slightly retarded during summer and yet may have continued later in the fall. From 2004–2007, high and low emergence numbers occurred in alternate years, suggesting a density dependent oscillation, but thereafter this pattern was no longer apparent. Nevertheless, one aspect of the data stands out clearly–even at ponds that lack fish, retain ample water throughout the year, and are apparently readily accessible to numerous adult *Anax* from April to September, emergence may fluctuate markedly from year to year. This alone might unpredictably push the selective advantage toward migration during years of poor success or toward overwintering in years of high success.

These data also clearly demonstrate another important variable that may affect the success of alternative life history strategies, i.e., the variation in relative success of early vs. late emerging larvae. Although the correlation of early emergence with subsequent production of overwintering larvae and, conversely, late emergence with southward migration of the emerged adults is probably much less rigid that Trottier [14] supposed, comparison of the timing of emergence and of southward migration strongly suggests that migratory individuals are primarily the imagos of late-emerging larvae; e.g., in most years the emergence minimum occurred in late July at KHMO (although in June in 2004 and not until mid-August in 2006), so migrants, most typically seen in late Aug. to Sept., probably emerged with the second peak. It is very likely that, when two clear peaks occurred, the early peak consisted primarily of larvae that had overwintered, while the later one included mainly larvae that had hatched in spring or early summer, many of which would migrate as adults. Thus a large early emergence peak probably indicates successful reproduction by the previous year’s early-emerging adults and also tends to bias emerging adults to lay eggs locally and produce larvae that overwinter and thus are exposed to unfavorable local or regional conditions, if any, in late summer through fall and winter; a larger late peak would have the opposite effects.

The influence of spring and summer rainfall on emergence success was marked (Table 1), despite the apparently small effect on conditions of the study pond. The additional water volume may buffer temperature and oxygen fluctuations, although this pond was probably less affected by these factors that many *Anax* breeding ponds. It may be that more successful emergence during wet years in nearby ponds increased recruitment of larvae to the study pond, although in that case we would expect greater effects later in the summer, rather than during early summer (peak 1) as was observed. Whatever the mechanism, however, seasonal rainfall clearly affected the number of adults emerging. On a geographic scale, of course, the amount and timing of rainfall is crucially important to reproductive success, as discussed below.

Data from RI also reveal striking variation in emergence timing and success, both within ponds between years and among ponds. Distances between ponds ranged up to about 60 km, which is within the daily flight range of adult *A*. *junius* [47], so a female could possibly oviposit in ponds spread over much of the entire study area in a few days. Smaller libellulid dragonflies evidently disperse, as a rule, no more than about 1–8 km from pond to pond [48, 49, 50], but some larger libellulids and macromiids in arid regions evidently may disperse over hundreds of km [51]. Only 7 pairs of RI ponds were located within 10 km of each other. Of these, both ponds of 6 pairs declined by 45–80% in exuviae per hour from 2004 to 2005 and both of the seventh pair increased slightly over the same years. Although obviously sparse, these data suggest that nearby ponds are likely to change in the same direction from year to year, thus reducing the value of bet-hedging within a small contiguous area.

Within the entire state, each annual sample included at least 14 sites (3 were identical in all 3 years [Fig 5] and 10 in addition were identical in 2004 and 2005 [S1 Fig]). Emergence success varied among years, with the overall emergence rate in 2005 about 35% less (20.1 exuviae per hour per pond) than in 2004 (31.6) and 2006 (30.2). Thus, on a regional scale, there should be stronger selection for long distance dispersal in some years than in others, although some buffering of this effect presumably occurs except under extreme conditions if oviposition is spread over several hundreds or thousands of km^2^. Also, despite the general year-to-year patterns, annual variation in emergence success was heterogeneous among ponds (Figs 5 and 6, S1 Fig). This suggests that even during years when regional emergence success is relatively high, female *A*. *junius* might benefit by ovipositing at multiple sites throughout the area, although this advantage may sometimes be less than it appears from the mean emergence numbers; in 2006 the rate of collection of exuviae from a single pond, Strathmore, was equivalent to the summed rates at all other ponds combined. Moreover, to the extent that a larger emergence peak is early at certain ponds in some years, and predominantly late in the same ponds in other years, the success of overwintering larvae vs. those that will become migrants as adults may fluctuate on a very local scale, independently of regional trends.

### Adaptation to hydroperiod

To a large extent, successful emergence of *A*. *junius* is constrained by hydroperiod. In most of the northeastern US and southeastern Canada, including the study sites discussed above (except Austin, TX), precipitation is more-or-less evenly distributed throughout the year (S2 Fig; this and most other information on U.S. precipitation and hydrology are from NOAA [52], USGS [53], U.S. Climate Data [54], and for Veracruz, MX, from ClimaTemps.com [55]. As result of snow melt and/or low levels of evapotranspiration, lentic water bodies are usually at their highest levels in late spring; low water levels occur in late summer and fall, although in years of near-normal rainfall and temperature, most perennial ponds support development of dragonfly larvae throughout this period. These circumstances ordinarily afford adequate time for overwintering larvae to emerge well before any serious deterioration of the environment. Many more of the offspring of adults that oviposit after migrating northward, typically arriving in April-June, will be unable to emerge until the late summer low-water period; pond condition is liable to deteriorate further as they mature, and some might, as adults, risk exposure to deleteriously cold weather before reproducing. For these, migration is likely to be a viable strategy, but the benefit of migration is certainly enhanced by the existence of predictably suitable destinations, i.e., fish-free with ample water and vegetation in warmer regions to the south of their natal area.

Precipitation regimes along the southern Atlantic Coast and throughout peninsula Florida provide such conditions. In most of this region rainfall, and consequently also the level of most lentic waters, is highest during summer, often with a distinct peak in July-September [56] (S2 Fig). This pattern is also present but less marked in the interior Southeast and along the northern Gulf Coast, where winter rains are more frequent (S2 Fig). Some females oviposit in near-coastal areas *en route* to their ultimate destination (May & Matthews, 2008); behavior at inland sites is probably similar but is poorly known.

**Fig 9:**
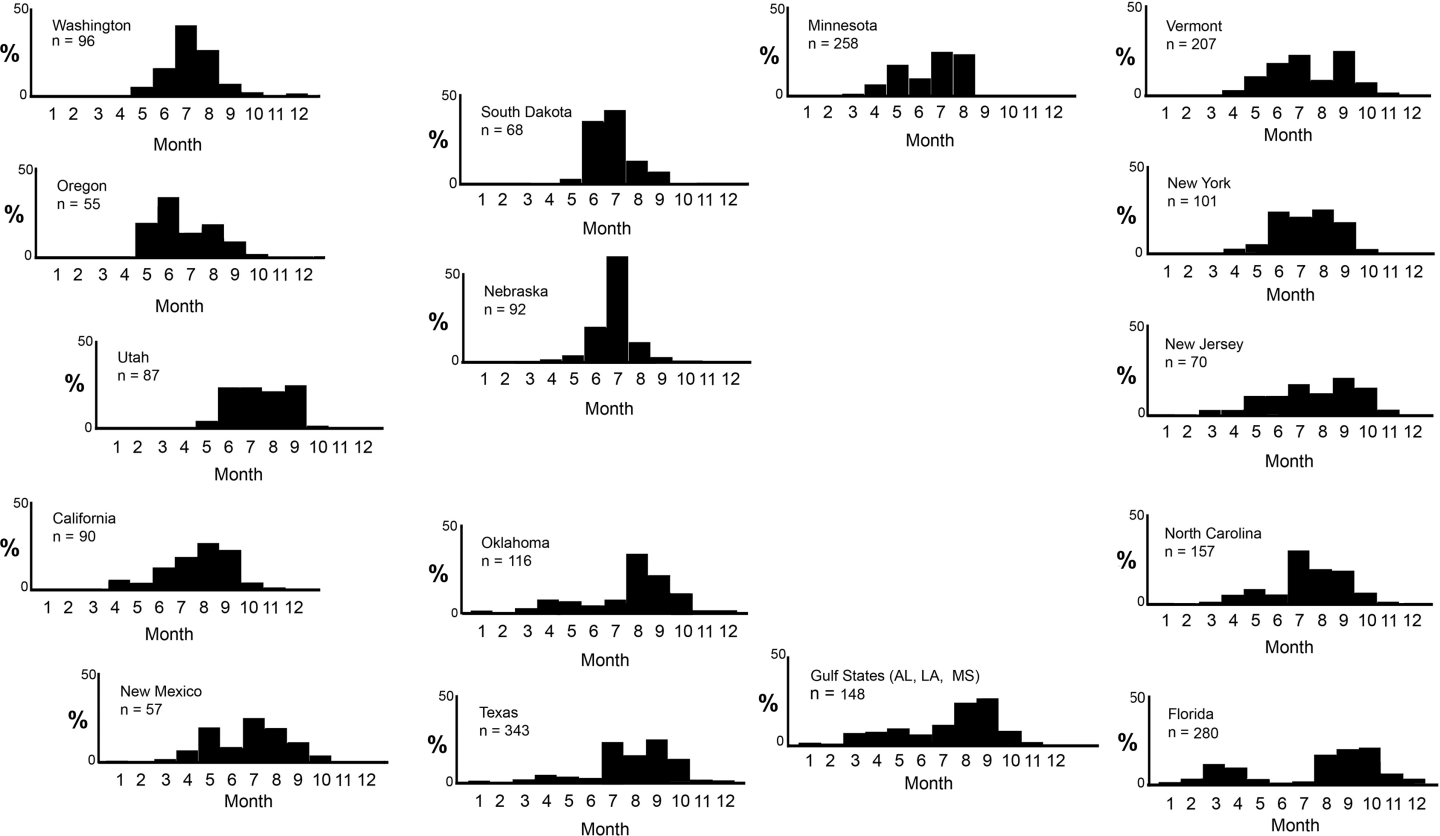
Monthly pattern of occurrence of adult *Anax junius* in various states of the United States. The percentage of the total number of records for each state is plotted against month. Only states for which at least 50 records were available are included, except that summed data for AL, LA, and MS are plotted on a single graph, labeled “Gulf States”, in order to include a sample size >50; patterns of occurrence were quite similar in each of the 3 states. Data are from Odonata Central [39], D. R. Paulson (pers. comm., 2016; DRP), and specimens in the Florida State Collection of Arthropods (FSCA). Histograms are arranged to indicate very roughly the geographic position of the corresponding U.S. state. Underlying data in S10 Table.

In the eastern U.S., August and September are the times of greatest southward movement of adult *A*. *junius*, [18] and these months plus October correspond to the period of greatest abundance of mature adults in Florida (Fig 8) [41]. Data for NC and AL, MS, and LA (combined data) from Odonata Central [39] and FSCA are similar (Fig 9), although spring and fall peaks and midsummer minimum are less pronounced in those states, perhaps at least in part because they extend further inland where winter temperatures are colder and the preponderance of summer rainfall is less. Large migratory swarms have been observed along the Gulf Coast in fall in the panhandle of Florida [57] and Alabama (K. Langin, pers. comm., 2006), and they almost certainly occur in Louisiana [58]. Relatively high levels of activity, including reproduction, occur also mainly in fall and winter in Veracruz and Yucatan, Mexico ([59]; JM, pers. obs. 2005; MLM, pers. obs. 2011). Most of Mexico has a summer wet season, so ponds and fresh marshes have high water in early autumn, with a low period typically in Feb.-May [55] (S2 Fig).

Final instar larvae are present in southern Florida at least from June until early April (N. Dorn, pers. comm., 2009; J. Trexler & R. Urgeles, pers. comm., 2008), which tends to corroborate the hypothesis that larval diapause does not occur there. The hiatus in May might reflect the difficulty of collecting larvae during this low water period when most aquatic fauna retreat to refugia that often are difficult to access, although these refugia often harbor high densities of fish [60], reducing their suitability for *A*. *junius* larvae (N. Dorn, pers. comm., 2009, 2016). As water rises in late summer, however, adult *Anax* can recolonize newly inundated ponds and other wetlands more rapidly than fish [61]. Thus, offspring of the influx of migrants in August might initially enjoy enhanced survival. Late spring and early summer may be periods of greater larval mortality from drought or predators, partly avoided by emergence in March and April.

### Migration in the West

The biology of *A*. *junius* in western North America is much less well documented than in the East, although southward mass migration flights have been observed in late summer and autumn near the coast and offshore [62, 63], as well as inland in California (Paulson, pers. comm., 2016), and in large swarms at the tip of Baja California Sur, MX, in November [64]. There is at least one report of a northward spring flight near Death Valley, east of the Sierras [65]. Kime [20] found what appeared to be a spring and a late summer cohort of larvae in lakes in eastern Washington, and Paulson noted that small numbers overwinter as larvae and emerge in spring each year in western Washington [8].

The ability of *A*. *junius* to thrive in western lakes contrasts with the usual situation in the East, where the most important predators of large Odonata larvae are probably centrarchid fish [9]. With one exception, the native range of centrarchids was east of the Rocky Mountains, and only in historic times have they become widespread in the western U.S. [66]. Probably as a consequence, *A*. *junius* in the West are evidently adapted to reproduce in permanent lakes [20, 67] as well as small ponds (D. Paulson, pers comm., 2000, 2016; S. Valley, pers. comm., 2016). Throughout most of the Pacific and Intermountain States, summers are extremely dry and in many places very hot, with many saline and hypersaline water bodies, which may often preclude successful development of a migratory generation in any but permanent or nearly permanent waters during most years, so reproductive success and selection for migration may differ somewhat in this region.

Likewise, the Central states, here including Minnesota and South Dakota through Texas (Fig 9), are characterized by a different range of climates and hydroperiod–obviously with a very strong north-south temperature gradient but all with roughly similar patterns of rainfall, with a peak in late spring and early summer followed by steady diminution in late summer and fall (S1 Table) [50, 52]. Observations in the northern areas suggest that adult activity is primarily concentrated in midsummer. In Oklahoma and Texas, however, although adults are present throughout the year, the great preponderance seem to occur from midsummer to October (Fig 9) [68], although Fig 7 shows that emergence in Texas can begin as early as late January and February. Some of the early observations of adults might represent residual migrants from the previous fall that overwintered in the south. The large numbers of adults later in the season may represent locally emerged individuals that escaped or avoided drying ponds as well as migrants from further north. Migration through the Central States is known to occur, e.g., [18, 22] but, again, has not been studied in depth. In Veracruz, MX, both adult migrants and larvae have been observed in September (JHM, MLM, pers. obs.).

## Conclusions

Several synergistic factors probably played a part in the evolution and maintenance of both overwintering diapause and migration in the life cycle *Anax junius*. Susceptibility of larvae to predation by fish means that successful reproduction must generally occur in fish-free water bodies (although adults may not be able to detect predaceous fish directly). Most such sites are relatively small and susceptible to drying. On the other hand, the necessity for floating or emergent vegetation for oviposition and as larval habitat requires that water be continuously present long enough, often for several years, for development of appropriate plants. This in turn means that, in any given year, there is a reasonable probability that larvae from eggs laid in a water body one year can emerge successfully, either in late summer of that year from eggs laid in spring or in the following year from eggs laid in summer. Our data, however, show that emergence success varies widely from year to year and site to site during both the spring to early summer and mid-summer to early fall periods, even in the absence of obvious catastrophic habitat collapse, so ovipositing adults must often place their offspring in habitats of unpredictable suitability. This may select for bet-hedging by females throughout the flight period by ovipositing in multiple ponds to spread the risk that any one pond might become unsuitable for larval development, although to our knowledge this behavior has not been documented. Migrating females, however, often have mature ovaries with many eggs and have been observed mating and ovipositing *en route* (JM, pers. obs.; May & Matthews, 2008; May, 2013). Moreover, widespread genetic homogenization across much of their range [15, 29] is highly suggestive that they distribute eggs widely at least during migration. These observations also strongly indicates that, unlike many other migratory insects [69] reproductive diapause is not part of the migration strategy of *A*. *junius*, despite its occurrence in many tropical non-migratory and short-distance migrant Odonata [13].

If a pond does dry up or become excessively warm and/or hypoxic, it is likely to be the larvae from eggs laid in summer that fail to emerge, because these events are most likely in late summer or early fall in the northern and western U.S. Adults that emerge in late summer may be faced with fewer fish-free oviposition sites, and individuals emerging very late in autumn (e.g., Fig 1) may have insufficient time to mature gametes and reproduce before the onset of cold weather, although active adults have been seen, on rare occasions, as late as November in VT (Fig 9) and December in NJ ([70]; MLM, pers. obs., 2001]). Larvae may also need to reach some minimum size for successful diapause [45]. In addition to the multiple circumstances that make successful reproduction in their natal region more problematic for late-emerging *A*. *junius*, the pattern of summer rainfall, particularly along the southern Atlantic Coast, Florida, and the Gulf Coast, normally assures fall migrants of abundant breeding sites in early fall. In addition, as fall progresses, prevailing northwesterly winds, especially associated with or following cold fronts, tend to assist southeastward flight [18, 47]. Thus while the potential detriment to newly emerged adults of remaining in the natal area increases later in the summer, the difficulty and uncertainty of locating a suitable place for reproduction at the endpoint of migration should be reduced at this time.

Direct quantification of egg and larval mortality and recruitment or of survival and fecundity of migrant adults currently is not practically obtainable. Nevertheless, based on the data available and the plausible assumption that, averaged over time and space, the fitness associated with “resident” and “migrant” behaviors is equal, a qualitative understanding is possible. The key feature of the selection regime leading to facultative migration in the system seems to be, as initially proposed, an intermediate level of uncertainty of larval survival, which may shift unpredictably among years and breeding sites to favor either overwintering by diapausing larvae or migration by late emerging adults. This maintains both strategies in the population, probably by virtue of phenotypic plasticity [71], since there is no evidence of genetic differentiation [15, 29, 30].

## Supporting information

S1 Fig Plots of *Anax junius* exuviae collected vs day of year for nine Rhode Island ponds surveyed in 2004 and 2005 only. Kittbig, Sailadump, and Skit were fish free, the remainder had fish; none were dry during the study. Underlying data in S6 Table.
(PDF)

S2 Fig Precipitation and temperature at cites representative of regions discussed in the text. Mean monthly precipitation (cm) is indicated by histograms, temperature (^o^C) by broken lines.
(PDF)

S1 Table Size distribution of larval *Anax junius* at PNRR during 2001–2005. Cf. Figs 1 and 2. Data are transcribed from field data sheets and refer to live larvae. Data are arranged chronologically but those from Patuxent Marsh (PM1, PM2) and Millrace Pond are listed separately. “Size” is maximum head width of each larva (mm), “Group” designates a group of larvae of the same head with collected on the same day, “Number” gives the size of the group (each larva is plotted separately in Figs 1 and 2). The “Comments” column primarily lists wing pad length (wp) and total body length (bl), measurements that were recorded but not used in plots because of the difficulty of measurement and/or variability owing to abdominal retraction and extension.
(XLSX)

S2 Table Number of *Anax junius* exuviae collected at PNRR during 2004–2005. Cf. Figs 1 and 2. Exuviae were collected as described in the text; an entry of “0” indicates that exuviae were sought but not found, “—”indicates that exuviae were not sought at the designated site on that date.
(XLSX)

S3 Table Size distribution of larval *Anax junius* at KHMO study pond during 2004. Cf. Fig 3. Data arranged as in S1 Table except that the “Comments” column is omitted.
(XLSX)

S4 Table Number of *Anax junius* exuviae collected daily at Kestrel Haven study pond during 2004–2013. Cf. Figs 3 and 4. Data are arranged by year (columns) and date (rows). At the end of each calendar month the total number for that month and the cumulative number to that point in the year is shown.
(XLSX)

S5 Table Number of *Anax junius* exuviae collected per person-hour as a function of day of year (DOY) and pond identity in Rhode Island in 2004. Cf. Figs 5 and 6. Abbreviated names of ponds appear in the first row. Dots indicate days when no collecting was attempted at a given pond, zeros indicate days when exuviae were sought but none were found.
(XLSX)

S6 Table Number of *Anax junius* exuviae collected per person-hour as a function of day of year (DOY) and pond identity in Rhode Island in 2005. Cf. Fig 6 and S1 Fig. Pond identification and data symbols as in S5 Table.
(XLSX)

S7 Table Number of *Anax junius* exuviae collected per person-hour as a function of day of year (DOY) and pond identity in Rhode Island in 2006. Cf. Fig 6. Pond identification and data symbols as in S5 Table.
(XLSX)

S8 Table Number of *Anax junius* exuviae collected in 2003–2005 at Austin, TX, pond, by day of year. Cf. Fig 7.
(XLSX)

S9 Table Number of adult *Anax junius* specimens in the Florida State Collection of Arthropods (FSCA) collected in Florida south of 29 N, by month. Cf. Fig 8. Data are ordered alphabetically by county; Volusia Co. is about 50% south of 29°N, all others listed are 90% or more south of that latitude.
(XLSX)

S10 Table List of *Anax junius* specimens from FSCA, Odonata Central (2016) and personal records and notes from D. R Paulson (pers. comm, 2016) for selected states in the United States. Cf. Fig 9. Data are ordered alphabetically by state, then by month within states. Note that most FSCA specimens are not georeferenced.
(XLSX)

S11 Table Rainfall for years of 2004–2013 and for selected months within those years, and numbers of exuviae collected at KHMO. Cf. Table 1. Total Exv–total number of exuviae collected for the year; Exv Pk1 –exuviae collected during the first peak of emergence; Exv Pk2 –exuviae collected during the second peak of emergence; Min Day–day selected as defining the boundary between emergence peaks 1 and 2.
(XLSX)
