# Supplementary figures and images for: Emergence phenology, uncertainty, and the evolution of migratory behavior in Anax junius (Odonata: Aeshnidae)
Source: PLoS One. 2017 Sep 8;12(9):e0183508. doi: 10.1371/journal.pone.0183508 (PMC5591007; doi:10.1371/journal.pone.0183508)

# EXUVIAE COLLECTED PER YEAR

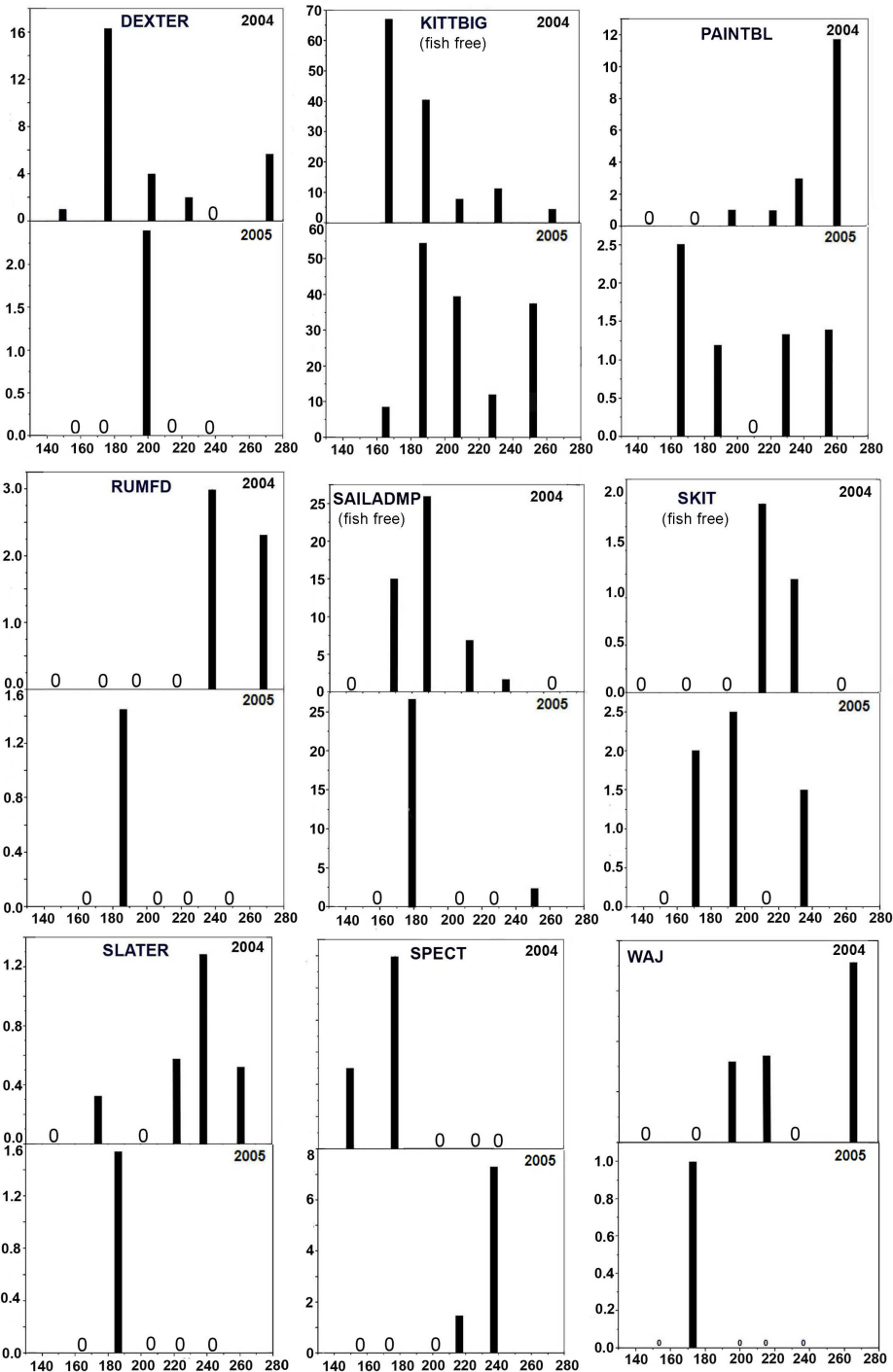

DAY OF YEAR

Supplement: S1 Fig — Kittbig, Sailadump, and Skit were fish free, the remainder had fish; none were dry during the study. Underlying data in S6 Table. (PDF) [file pone.0183508.s001.pdf]

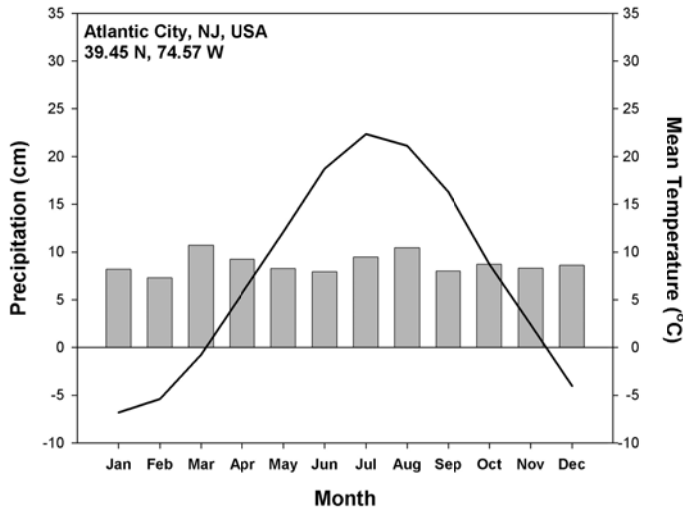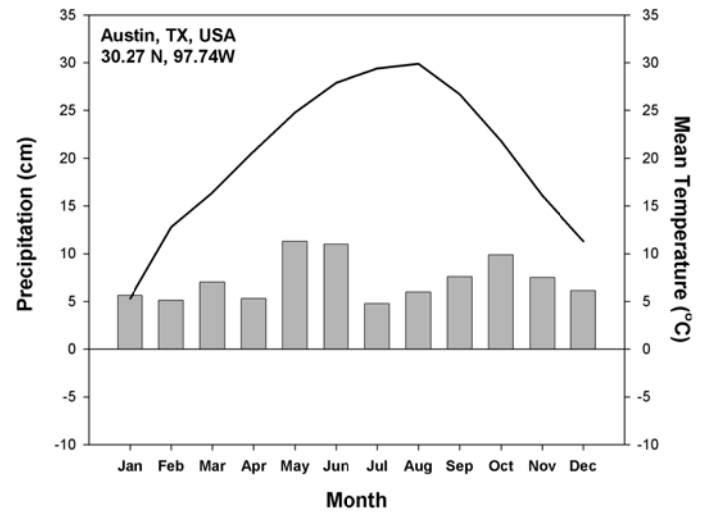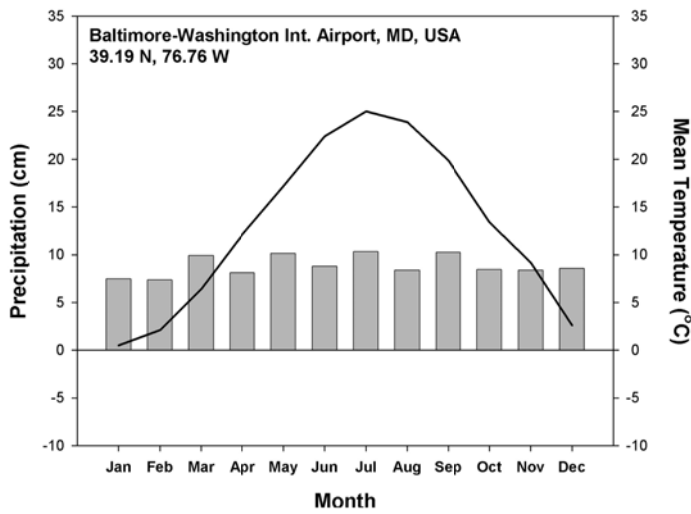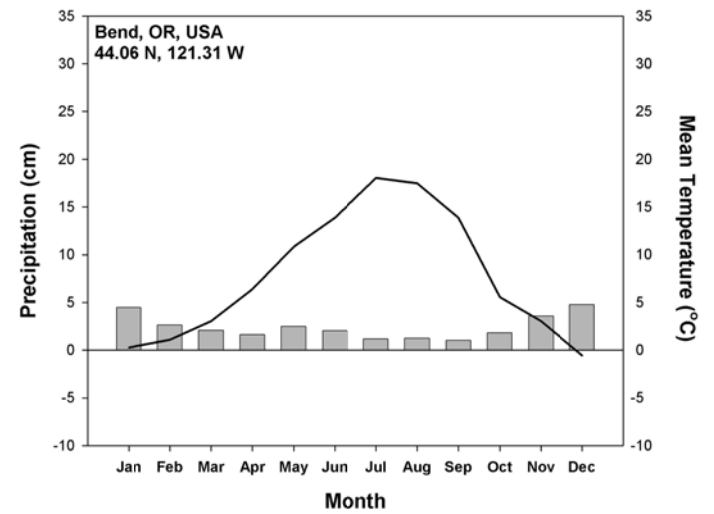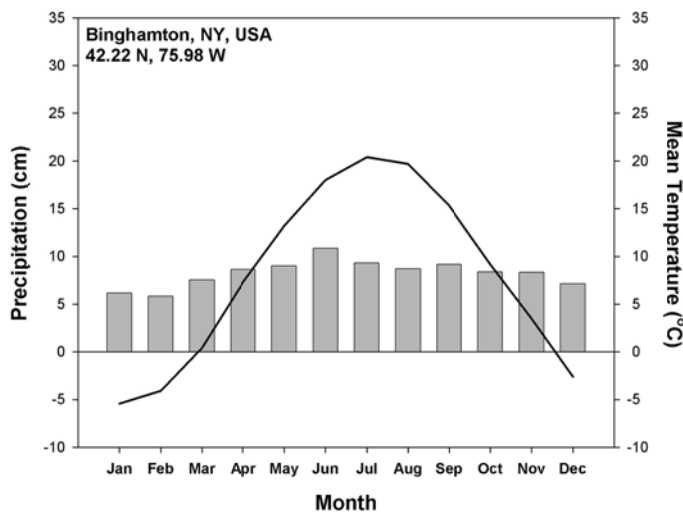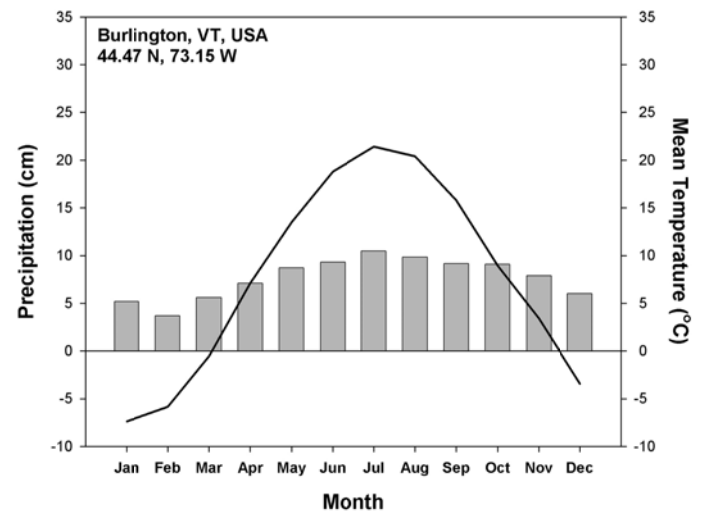

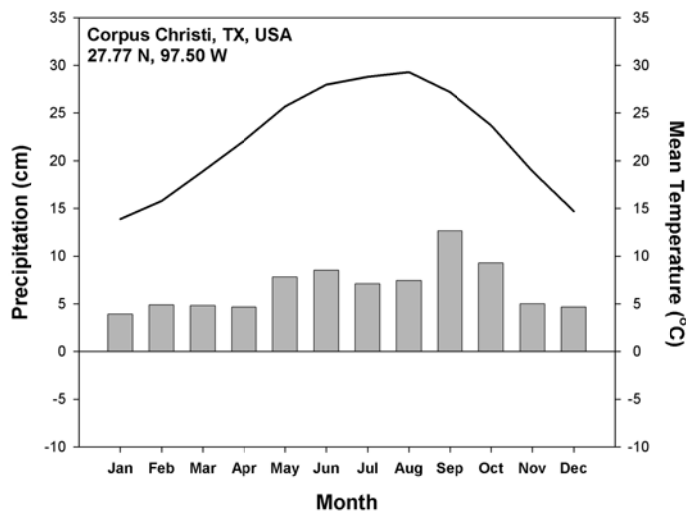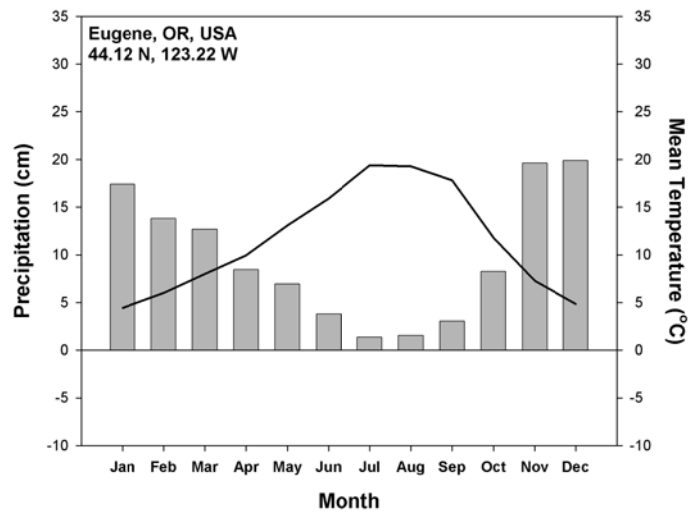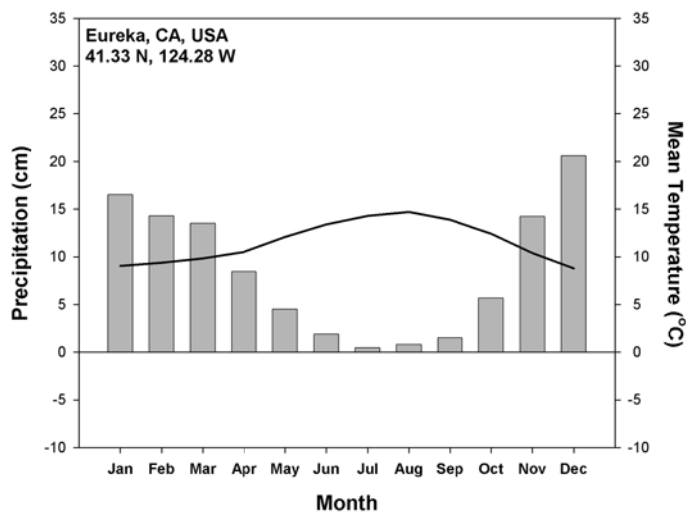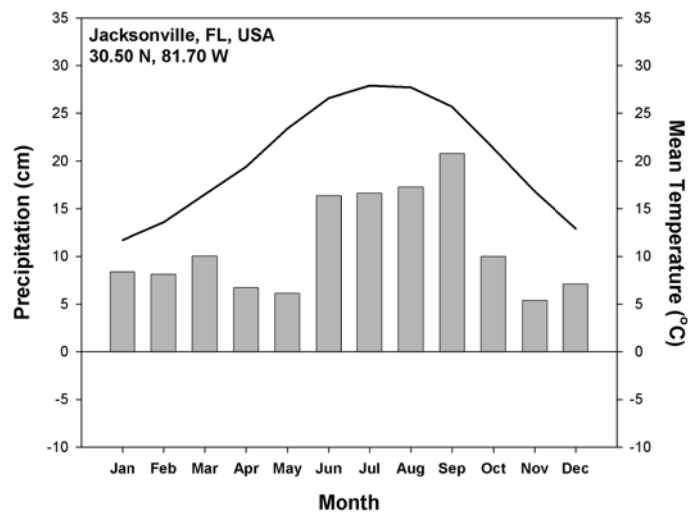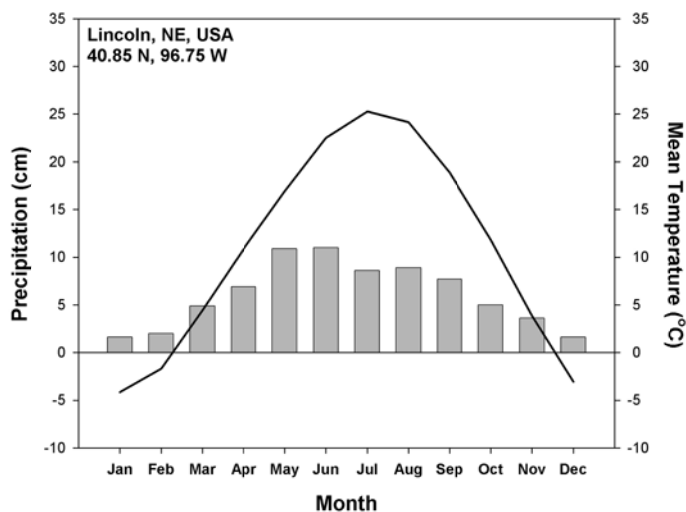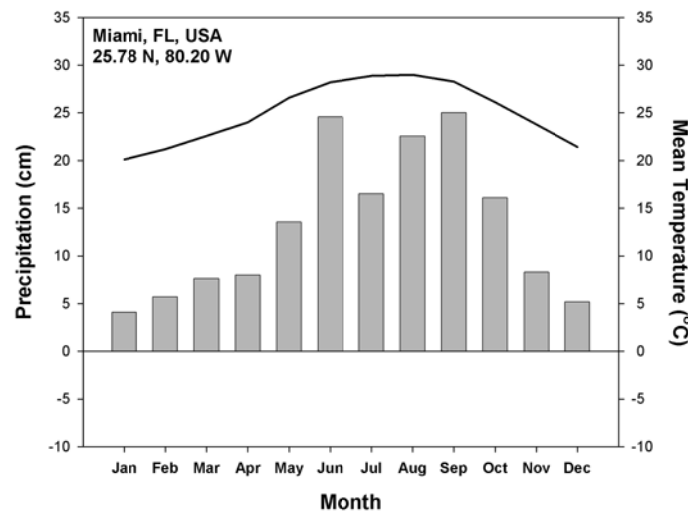

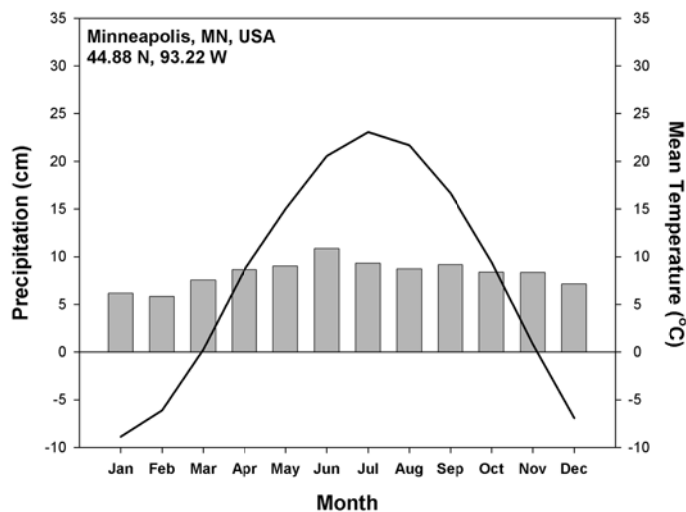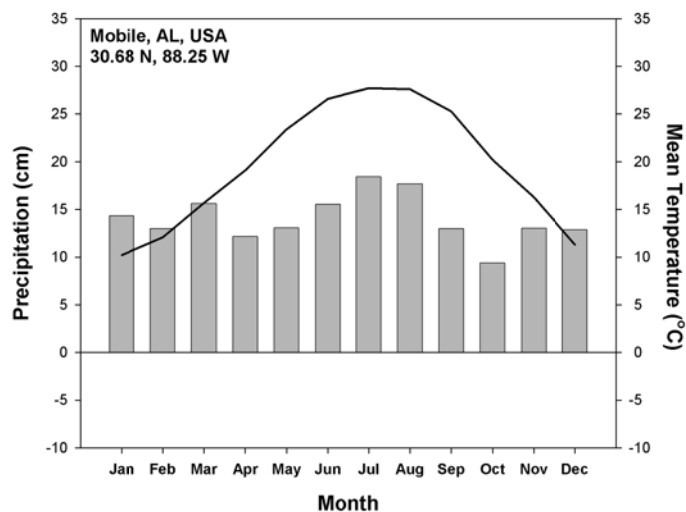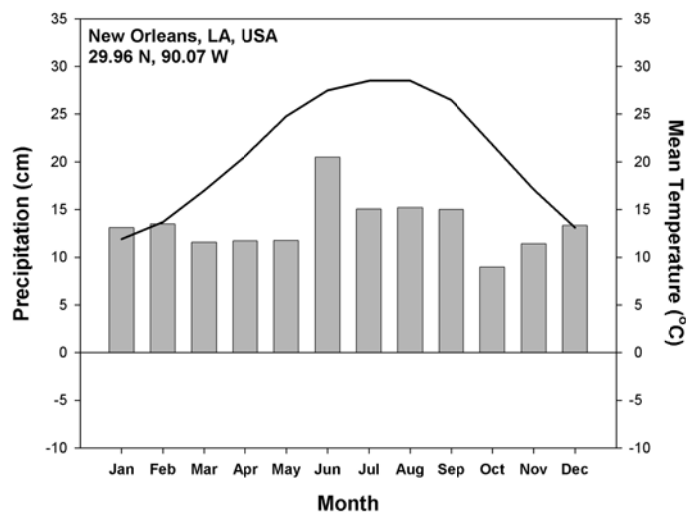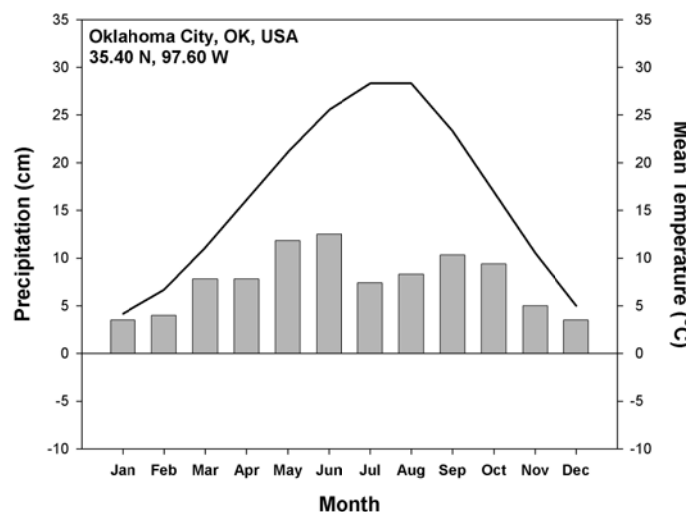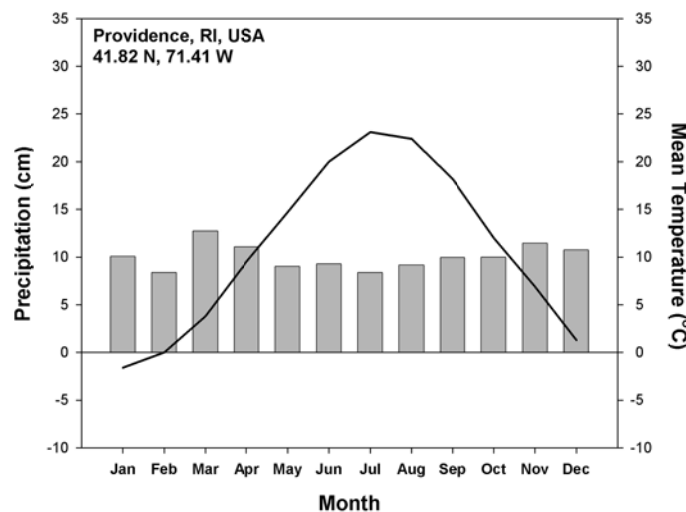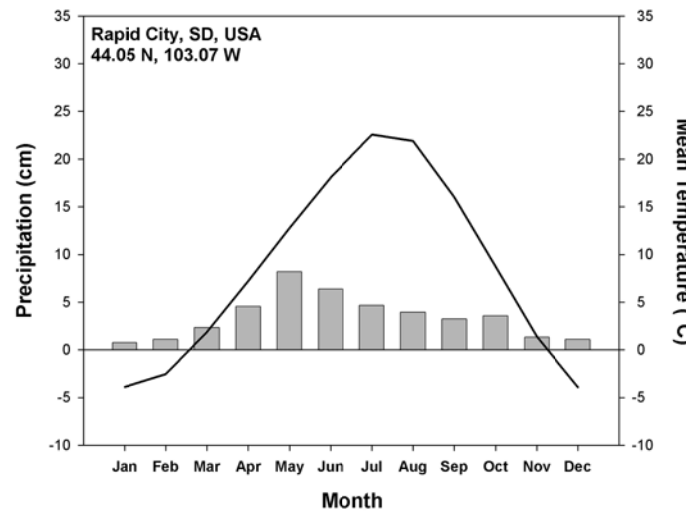

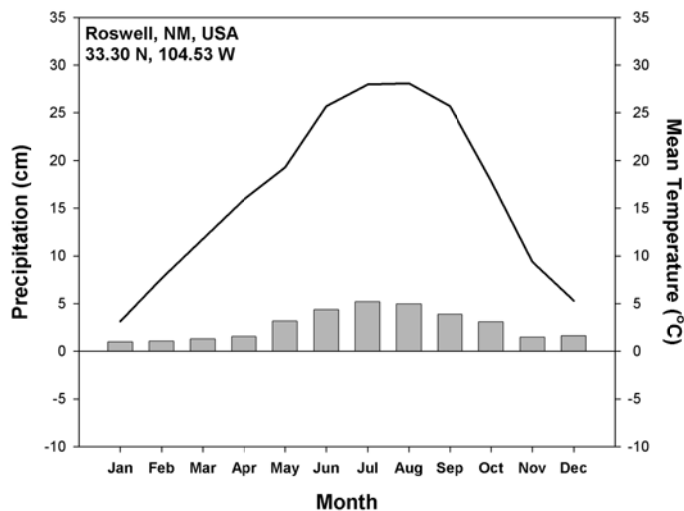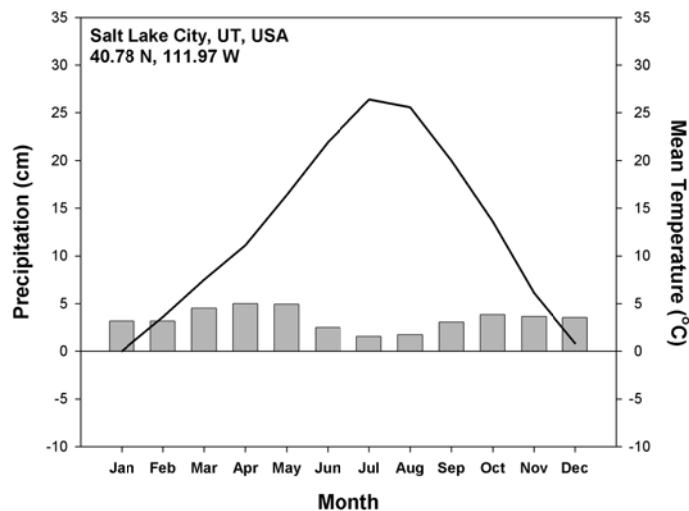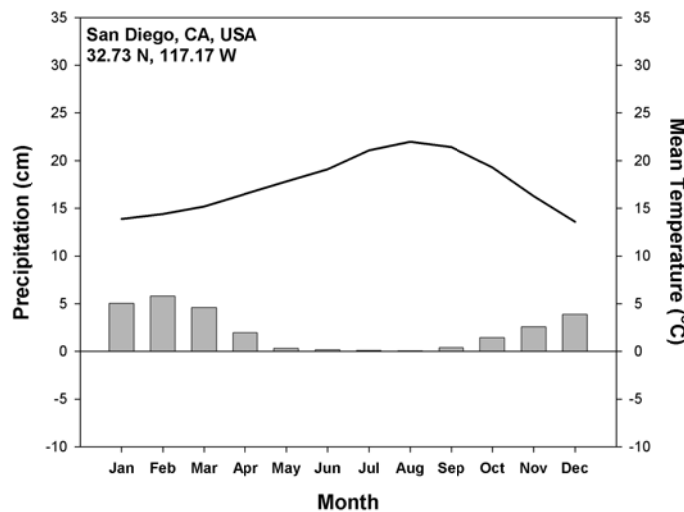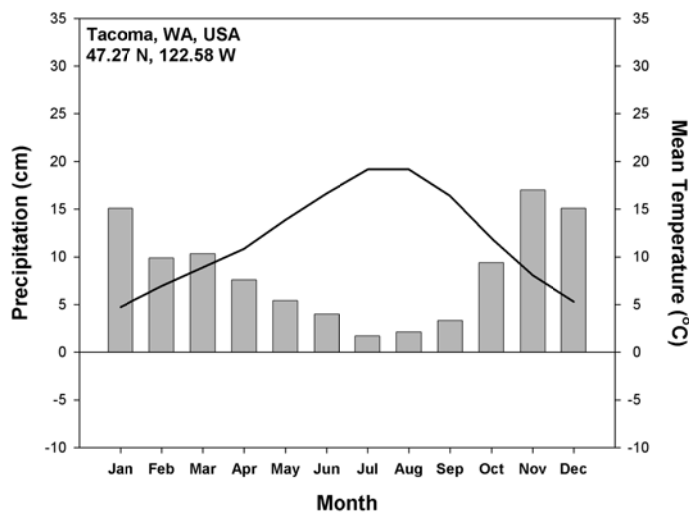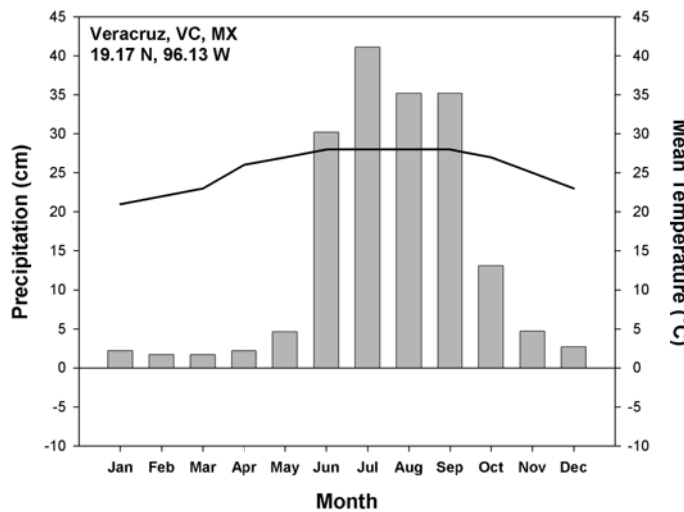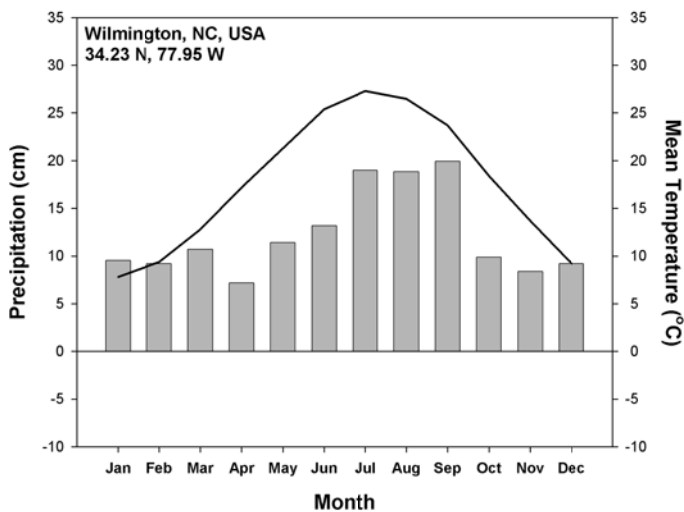

Supplement: S2 Fig — Mean monthly precipitation (cm) is indicated by histograms, temperature (oC) by broken lines. (PDF) [file pone.0183508.s002.pdf]
